# Country ownership and sustainability of Nigeria’s HIV/AIDS Supply Chain System: qualitative perceptions of progress, challenges and prospects

**DOI:** 10.1186/s40545-018-0148-8

**Published:** 2018-09-10

**Authors:** Ademola Joshua Itiola, Kenneth Anene Agu

**Affiliations:** West African Postgraduate College of Pharmacists, Lagos, Nigeria

**Keywords:** Country ownership, Sustainability, Supply chain system, Human resource, Health financing, Coordinating structures, Nigeria supply chain integration project

## Abstract

**Background:**

The emergency response phase to HIV epidemic in Nigeria and other countries saw to the deployment of donors’ resources with little consideration for country ownership (CO) and sustainability. The progress that has been made in the fight against the pandemic has however precipitated a paradigm shift towards CO and sustainability. With the decline in donors’ funding, countries must continually evaluate their readiness to own and sustain their HIV response especially the supply chain system (SCS) and bridge any observed gaps. This study assessed the current understanding of CO and sustainability of Nigeria’s HIV/AIDS SCS, established progress that has been made, identified challenges that may be hampering CO and possible recommendations to address these challenges. It also explored opportunities that the country can leverage on.

**Methods:**

We conducted a cross sectional descriptive study through semi-structured interview of twelve purposefully selected key informants involved in HIV/AIDS supply chain management. Transcribed qualitative data were analyzed using a thematic approach.

**Results:**

Among other submissions, respondents acknowledged that CO involves non-government stakeholders. Key CO and sustainability achievements were: development of national strategic plans and policy documents, establishment of coordinating structures, allocation of funds for some logistics activities at the state level and payment of salaries of government staff, institution of pre-service training, use of logistics data for decision making and the unification of the hitherto parallel HIV/AIDS supply chains. Challenges included: inadequate domestic funding, bureaucratic bottlenecks and inadequate manpower at the health facility level. Respondents recommended more political commitment and increased government funding, exploration of alternative sources of funding, improved accountability, effective healthcare workforce planning and local manufacture of HIV commodities. Existing structures and programmes that the country can leverage on included: Nigeria Supply Chain Integration Project, National Health Insurance Scheme and the private sector.

**Conclusions:**

Nigeria has made some progress towards achieving CO and sustainability. The country however needs to address financial and human resource gaps through innovative resource mobilization and effective workforce planning. As other countries plan for CO and sustainability, it is important to secure political buy-in and adopt a working definition for CO and sustainability while resource mobilization and workforce planning should be prioritized.

**Electronic supplementary material:**

The online version of this article (10.1186/s40545-018-0148-8) contains supplementary material, which is available to authorized users.

## Background

According to World Health Organization (WHO), approximately 35 million deaths have been recorded globally since the beginning of Human Immunodeficiency Virus/Acquired immune Deficiency Syndrome (HIV/AIDS) pandemic [1]. Seventy percent of the 37 million people that were globally infected as of 2015 were from Sub-Saharan Africa [1] with Nigeria ranking as the country with the second highest number of infected people and the highest number of infected children [2–5]. These alarming statistics make HIV/AIDS a disease of public health importance. In order to curb the scourge of this epidemic, a lot of research and investment had gone into fighting the pandemic [6]. These efforts have yielded positive results as the number of AIDS-related deaths and new infections has consistently reduced over the last decade [7]. HIV positive individuals are now living longer and the infection is now a chronic condition that requires long term management [8]. To exemplify the progress that has been made in Nigeria, the prevalence of HIV dropped from a peak of 5.8% in 2001 to 2.9% in 2014 [9–11]. The numbers of new HIV infections and AIDS-related deaths have also decreased by 21% and 6% respectively since 2010 although treatment gap still stands at 70% [12].

HIV response especially in low- and middle- income countries has been largely donor driven (proportion of donor contribution has been estimated to be as high as 97%) [1, 6] with the United States Government through President’s Emergency Fund for AIDS Relief (PEPFAR), the Global Fund and the World Bank standing as the major donors [13, 14]. As of 2012, domestic funding in Nigeria stood at 23% with PEPFAR and the Global Fund accounting for 43% and 33% of the total HIV spending respectively [15]. During the emergency response phase to this pandemic, resources were deployed with little consideration for Country ownership (CO) and sustainability largely due to the need for a swift response to address the ravaging epidemic in a manner similar to that of natural disaster response [16, 17]. However, with the progress that has been made so far in the fight against the pandemic, there is a paradigm shift towards CO and sustainability as stressed in 2005 Paris Declaration, the Accra Agenda for Action released in 2008 and the Busan Partnership for Effective Development Cooperation (2011) largely due to decline in donor funding [16, 18, 19]. With CO, countries are expected to have a national plan that prioritizes cost effective interventions; have institutions (both public and private) that are manned by individuals with requisite skills and competencies to drive interventions; lead the process of accountability to the international community and ultimately fund their HIV response [20]. Central to the attainment of CO and sustainability as well as health developmental goals is a strong health system that is based on the six building blocks: leadership/governance, healthcare financing, health workforce, medical products, technologies, information and research and service delivery proposed in the WHO's health systems framework [21]. These building blocks must be strengthened to achieve the best health outcomes [21]. In spite of the support from international donors, the health systems in most developing countries are still adjudged to be weak [22].

The decline and withdrawal of donor funding for certain services (donors no longer support certain laboratory investigations such as chemistry and some hematology tests in Nigeria) makes it imperative now more than ever before for countries to own and fund their HIV response including supply chain management (SCM) of HIV/AIDS products in order to prevent loss of the health gains that have accrued over the years [6, 13, 23, 24]. Experience from South Africa, Botswana, Namibia and Eastern Caribbean countries that had transitioned out of PEPFAR support suggests that transition must be well managed if gains will not be reversed. In South Africa, transition is reported to have resulted in loss of 50,000–200,000 patients from care while some high impact prevention activities were left unfunded [19]. Romania also witnessed an increase in HIV prevalence among people who inject drugs from a baseline of < 2% in 2006 to 53% in 2013 with Global Fund transition [25]. Similar trend was observed in Serbia and Belarus [25].

With the pivotal role of an effective HIV/AIDS supply chain system (SCS) in service delivery and given that over 50% of healthcare expenditure goes into medicines procurement and supply management, ensuring that a robust and sustainable supply chain system is in place is essential for uninterrupted service delivery [26–28]. (See Additional file 1 for an overview of Nigeria’s HIV/AIDS SCS and Additional file 2 for overview of Nigeria’s healthcare system). Nigeria and most countries in sub Saharan Africa were not able to achieve most of the Millennium Development Goal targets and health products supply chains have been identified as the weakest link [28–30]. As Nigeria implements the WHO-recommended test and treat policy, there will be more pressure on the SCS hence the need to put appropriate system strengthening structures in place [31, 32].

Countries (including Nigeria) have made some progress with respect to CO despite challenges. It is therefore important to explore the current understanding of CO and sustainability, document the most updated progress that has been made in Nigeria, identify challenges that may be hampering CO as well as potential recommendations and opportunities that the country can leverage on. This study examined these issues as it relates to HIV/AIDS Supply Chain System.

## Methods

Twelve purposefully selected key informants (KIs) involved in supply chain management (SCM) of HIV/AIDS products were interviewed using an interview guide that comprised of 7 broad questions (see Additional file 3). The development of the interview guide was largely informed by the United States Interagency Paper on Country Ownership [20] as well as the study objectives. The key informants were selected because of their involvement in HIV/AIDS SCM either at the policy or service delivery level leveraging on the lead researcher’s experience within the HIV/AIDS SCM space. Six Directors of Pharmaceutical Services (one from each geopolitical zone of the country in order to allow for some degree of country representativeness) were chosen because they oversee all HIV/AIDS SCM activities within the states. The interview guide was piloted with two stakeholders prior to use in the actual study. Questions were modified accordingly based on findings from the pilot. Six of the interviews were conducted face to face while the rest were conducted via a cell phone. The tight schedule of six key informants made it difficult to schedule face to face interview and this necessitated conducting these interviews through this medium. The qualitative data were transcribed, coded and analyzed using a thematic approach. Themes were grouped using WHO health systems framework and the results were discussed along the study objectives. Both published and grey literatures were also reviewed to validate findings in addition to triangulating information provided by the key informants [10, 13, 22, 24, 33–41]. It is noteworthy that there is a quantitative component to this study which will be published in a separate paper.

## Results

Age of key informants ranged between 35 and 59 years with most (83.4%) of the respondents between 45 and 64 years. Most of the respondents (91.7%) were male. Respondents’ years of experience in HIV/AIDS SCM were between 2 and 11 years. Half of the respondents had between 6 to 10 years of SCM experience while the same proportion had Bachelor’s Degree as their highest level of qualification. Most (75.0%) of them work in the public sector. The interviews lasted for between 14 to 70 min with an average of 34 min per interview. All interviews were completed in August 2016. Respondents include: Director of Pharmaceutical Services (six), Logistics leads at the Federal Ministry of Health and national Agency for the Control of AIDS (two), Staff of international donor agency (one), implementing partner (one), logistics firm (one) as well as health care worker (one). Further details are available in Table 1. The themes identified were grouped under 6 broad headings using WHO health systems framework (see Table 2 for summary of findings and Additional file 4 for list of selected quotes).

**Table 1:** Demographic Characteristics of Key Informants

Variable	Categorization	n (%)
Age	35–44 years	2 (16.6%)
	45–54 years	5 (41.7%)
	55–64 years	5 (41.7%)
	Total	12 (100.0%)
Gender	Male	11 (91.7%)
	Female	1 (8.3%)
	Total	12 (100.0%)
Years of Experience in SCM	1–5 years	3 (25.0%)
	6–10 years	6 (50.0%)
	11–15 years	3 (25.0%)
	Total	12 (100.0%)
Sector of Engagement	Public	9 (75.0%)
	Private	3 (25.0%)
	Total	12 (100.0%)
Job Title	Director of Pharmaceutical Services	6 (50.0%)
	Associate Director	1 (8.3%)
	Head HIV/AIDS Logistics Unit	1 (8.3%)
	Head, Supply Chain Management unit	1 (8.3%)
	Commodities Logistics Manager	1 (8.3%)
	Senior Specialist	1 (8.3%)
	Head of Department, Pharmacy Unit	1 (8.3%)
	Total	12 (100.0%)
Educational Qualification	Bachelor Degree	6 (50.0%)
	Fellowship	1 (8.3%)
	Masters	5 (41.7%)
	Total	12 (100.0%)
Residence, Working	Abia	1 (8.3%)
	Abuja	6 (50.0%)
	Delta	1 (8.3%)
	Nasarawa	1 (8.3%)
	Oyo	1 (8.3%)
	Sokoto	1 (8.3%)
	Yobe	1 (8.3%)
	Total	12 (100.0%)
Interview Duration (in minutes)	11–20	1 (8.3%)
	21–30	4 (33.3%)
	31–40	4 (33.3%)
	41–50	2 (16.7%)
	51–60	0 (0.0%)
	61–70	1 (8.3%)
	Total	12 (100.0%)
	Average	34

**Table 2:** Summary of Key Findings

WHO Health System Building Block	Themes	Understanding	Key Findings		Recommendations	Opportunities
			Achievements	Challenges		
Leadership/Governance	Government Leadership, Government Commitment, Political Will, Continuity, Strategic Plans and Policies, Coordination and Coordinating Structures	Country ownership (CO) involves government providing strategic direction, showing more commitment and taking up more responsibility in the management of its HIV response	Government is demonstrating CO through the takeover of HIV/AIDS services in 2 states	Nigeria’s HIV response has been largely donor driven while lack of political will and poor leadership are some of the factors hampering CO in Nigeria	Advocacy to top government officials can engender CO	
		CO does not necessarily mean government should be responsible for direct implementation; CO also involves multiple stakeholders	Nigeria has strategic plans and policies that will engender CO	The Strategic Plan may not have the appropriate implementation plan	Respondents feel donor support should still continue for a while	
		Plans and Policy frameworks are crucial for attainment of CO	Nigeria has made significant progress when it comes to coordination	Paucity of funds may affect optimal functioning of Logistics Management Coordinating Unit (LMCU)	Government should maximize the presence of donors to strengthen other aspects of the healthcare system	
	Donor Support	Supply Chain System (SCS) must be adaptable	General in-country HIV coordination structures include, National Agency for the Control of AIDS (NACA) and National AIDS and STI Control Program (NASCP) at the national level, SACAs and LACAs at state and LGA levels respectively	Attention of donors may shift to other interventions or countries	Systems (possibly electronic) must be put in place to ensure accountability and the current administration appear to be pursing this vigorously	
	Transparency and Accountability, Public-Private Partnership	Sustainability involves continuity of program after termination of donor(s)’ support	National Product and Supply Chain Management Programme (NPSCMP), Procurement and Supply Management Technical Working Groups (PSM TWGs) and LMCUs are the Supply Chain Coordination Structures in the country (covers other programs outside HIV)	Transparency and Accountability in government is poor as procurement processes are sometimes not followed	Government must demonstrate willingness to be accountable in order to encourage private sector funding	
		Some level of program modification such as patients paying for HIV/AIDS services may be necessary for sustainability	There may be need and it appears plans are underway to establish LMCUs at local government level	Some shady deals may also be going on within the government settings	The private sector and partner can be of help to government in addressing certain challenges	
			Nigeria has a near-perfect Public Procurement Act	NACA may not have all the necessary structures for grant management	Government may need to move away from running the supply chain and donors may be more comfortable working through the private sector due to better accountability	
	Prevention				Government needs to prioritize prevention activities to reduce new infections	
Healthcare financing	Domestic Funding, Bureaucratic Bottlenecks, Alternative Funding Sources	Funding of a large percentage of a country’s HIV response by the government is a marker of CO	There was a time a special intervention fund in form of President's comprehensive response plan (PCRP) was set up by government (this may still be on under a new name) and there are plans to increase funding	Inadequate domestic funding is the biggest challenge hampering CO which in turn has affected the ability of the government to bridge the gap left by donors.	Payment of subsidized fee for HIV services is recommended as a means of engendering sustainability and improving adherence	Private sector is involved in Nigeria’s HIV response and can be a possible source of financing Nigeria’s HIV response-even though government funding might just be sufficient
			PCRP has an element of fund mobilization from the private sector	The challenge of funding is further compounded by the poor economic situation of Nigeria	Private companies should not be compelled by law to fund HIV response but can be incentivized to do so	
			There is evidence of sub-national fund allocation	There is evidence to show that funding for certain activities is being withdrawn	States can support HIV programme through their drug revolving fund (DRF)	
			There is visibility into funding of donors and government only bridges gap	Out of pocket payment by HIV patients may not be a viable option for domestic financing	Expansion of National Health Insurance Scheme (NHIS) coverage to the informal sector is also important	
				Funds budgeted by government are sometimes not released at all and when released are either not timely or not in full or both	High level advocacy to the relevant stakeholders can catalyze sub-national funding allocation	
				Available resources may hinder implementation of test and treat policy	Government funding should extend beyond procurement but must also cover other supply chain expenses such as port clearance, distribution e.t.c.	
					Significant co-funding by government may improve co-planning with donors	
Health workforce	Human Resource for Health, Training and Mentoring, Capabilities and Behavioural Disposition		Most of the staff at the lower levels are Nigerians employed by government	There is adequate capacity at the national level (especially in the private sector) but not yet at the sub-national level	Advocacy to relevant authorities can help reduce transfer of trained staff to sites where there skills may not be needed	Private sector is involved and can be used for capacity building
			Donor intervention has contributed to capacity building for healthcare workers	There is dearth of manpower for service delivery and the current poor economic situation may not allow for recruitment in the immediate future, Capacity also needs to be built on an ongoing basis	Workers need to be motivated for optimal performance through regular salary payment, absorption of ad hoc staff under appropriate terms of service	
			Pre-Service Training has been instituted in the schools of Pharmacy, Laboratory Science and Health Technology as a means of addressing the human resource capacity gap	Attrition of trained staff is a big challenge with remuneration differential contributing to this attrition as well as inequitable distribution of healthcare professionals	There may be need to prioritize certain professionals for some logistics roles	
				Capacity gaps exist in areas such as quantification and program management at the state and national levels	There may be need for professional recognition of logisticians and special remuneration for those performing logistics functions	
				Poor attitude to work is impacting negatively on CO	Task shifting will be of help in addressing the human resource challenge	
					Clear performance benchmark can address some of the attitudinal challenges	
					Proper planning and mentorship can help address capacity gap	
					Capacity of government officials need to be built as some changes are dependent on them	
					Government can also attract best brains from the private sector to improve public sector performance	
					It might be helpful to institutionalize logistics as a professional postgraduate programme in Nigeria	
Medical products, technologies	Local Manufacturing, Unification and Integration, Infrastructure, Public-Private Partnership	Local production HIV commodities is a marker of CO	Nigeria has a unified HIV/AIDS Supply Chain which leveraged on existing government structures		Even though there is no local production of ARVs, local production has the potential of fostering price reduction and continuous product availability	Nigeria is in the process of integrating supply chain systems across multiple programme areas and this should enhance overall program effectiveness and efficiency
			Nigeria has warehouses that can be used for commodity storage			The LMCU is the state-level technical arm for integration
			Warehouse-in-a-Box is planned to be managed through a public- private partnership arrangement			
Information and research	Data availability, Data Quality and Data for Action		The LMCUs have increased visibility into happenings in other program areas and use of data for decision making	Quality of data in other programme areas may need more improvement	Electronic data capture system will help improve data quality	
			Logistics data is available and of improved quality, there may however be need to put appropriate infrastructures in place			
			Logistics data is now being used for advocacy and decision making			
			LMCU is doing a lot on the use of data for action and is also serving as an avenue to stimulate improvement of the data quality for other program areas			
Service delivery	Physical infrastructure Access, Integration		One of the major contributions of government towards CO is making available HFs for service delivery and payment of staff salaries		Infrastructures such as vehicles and even road network need to be improved upon	
			Donor support has contributed significantly to infrastructural upgrades. Donors and partners also provided infrastructural donations to LMCU		Government should consider integration of health services as this can potentially reduce the reporting burden on healthcare workers and reduce stigmatization	
			New sites are being activated to improve access but more needs to be done to improve coverage			
			Stigma has reduced while treatment acceptance has improved			
			Private health facilities are being used to improve access			

### Leadership and governance

Leadership and governance is critical to providing overall strategic guidance and policy regulation for the health system. Due consideration must be given to system design and accountability while providing leadership and governance oversight [20].

#### Government leadership, government commitment, political will

Respondents mentioned that CO involves government providing strategic direction, showing more commitment and taking up more responsibilities in the management of HIV response (Respondent (R)-1; R-3; R-4; R-5; R-7; R-8; R-11; R-12). CO is equally all-inclusive and involves several stakeholders including the private sector, communities and people living with HIV/AIDS (R-2; R-12). CO does not necessarily mean government should be responsible for direct implementation (R-2; R-12). The need for an adaptable system that flexibly imbibes latest innovations was acknowledged (R-9; R-12).

Nigeria has demonstrated political commitment by making health facilities available for service delivery and paying staff salaries (R-8). In addition, government has taken over programmme implementation in Abia and Taraba States (R-2).

Lack of political will and poor leadership were highlighted as major hindrances to CO in Nigeria (R-2; R-4; R-9; R-10; R-12) while advocacy to top government officials can help engender CO (R-1; R-2).

(See Quotes 1.1.1–1.1.7).

#### Continuity

From responses provided, sustainability will entail continuity of Nigeria’s HIV programme after termination of donors’ support (R-1; R-2; R-4; R-7; R-8; R-12) (See Quote 1.2.1). One of the respondents remarked that the only way the HIV programme can be sustained in the country is for patients to start paying subsidized fee for their care and treatment (R-3) (See Quote 1.2.2).

#### Strategic plan and policies

Strategic plans and policy frameworks are crucial for the attainment of CO and that the country has a National Strategic Health Development Plan (2010–2015) even though it appears that the plan for 2016–2020 is not yet officially available (R-1; R-2; R-4; R-6; R-7; R-8; R-9; R-12) (See Quotes 1.3.1.0–1.3.1.1). Other policies and act that are in place are: The National Supply Chain Policy for Pharmaceuticals and Other Healthcare Products and the National Procurement Act (R-12). Respondent however decried poor implementation of policies and enforcement of the act (R-11; R-12) (See Quote 1.3.2).

#### Coordination and coordinating structures

Key informants remarked that Nigeria has made significant progress with respect to coordination (R-1; R-10; R-11; R-12). The overarching HIV programme coordination structures include: National Agency for the Control of AIDS (NACA) and National AIDS and STI Control Program (NASCP) at the national level as well as State Agencies for the Control of AIDS (SACAs) and Local Government Agencies for the Control of AIDS (LACAs) at state and LGA levels respectively (R-1; R-2; R-3; R-4; R-7; R-8). HIV/AIDS SCM-specific coordination structures mentioned include: National Product Supply Chain Management Programme (NPSCMP) at the national level, Procurement and Supply Management Technical Working Groups (PSM TWGs) at the national, regional and state levels and Logistics Management Coordinating Unit at the state level (R-1; R-2; R-4; R-5; R-11; R-12). These HIV/AIDS SCM coordination structures are not just limited to HIV/AIDS but extend to other disease programme areas. One of the respondents noted that there may be need (and it appears plans are underway) to establish LMCUs at local government level since the primary health centres are under the direct supervision of local government authorities (R-5). Paucity of funds may be affecting the optimal functioning of LMCUs in some states (R-6).

(See Quotes 1.4.1–1.4.5).

#### Donor support

Respondents acknowledged that Nigeria’s HIV response has been largely donor driven and that the support had contributed significantly to capacity building for the locals as well as in-country infrastructural upgrades (R-2; R-4; R-6; R-7: R-10; R-11). They think that donor support should continue for a while as Nigeria may not have the requisite funding required to take full ownership (R-2; R-5; R-7). Some of the respondents noted that the country should maximize the presence of donors by using “saved” resources to strengthen other aspects of the healthcare system that the donors are not currently supporting. They equally called for the integration of best practices from donor–funded programmes into the general healthcare system (R-4; R-11; R-12). Finally, a respondent noted that the attention of donors may shift to other interventions or countries hence the need for stronger government commitment (R-7).

(See Quotes 1.5.1–1.5.4)

#### Transparency and accountability

Two national-level respondents noted that Nigeria has a near-perfect Public Procurement Act (PPA) that may only need minor amendments (R-11; R-12). Implementation of the Act was however identified as the major challenge; transparency and accountability in government was adjudged to be poor as procurement processes are sometimes not followed (R-3; R-6; R-9; R-10; R-12). Comments from some of the respondents suggested that there may be some corrupt elements perpetrating shady deals in the government (R-5; R-8; R-11). Specific reference was made to the alleged embezzlement of donor funds by Nigeria’s Department of Planning Research and Statistics (DPRS) (sub-recipient to NACA) and NACA’s admittance that the organization does not have the requisite structures for financial oversight (R-3; R-4).

Respondents suggested that systems (possibly electronic) must be put in place to ensure accountability and it appears that the current administration in the country is pro-accountability and transparency (R-6; R-9; R-12). Response from one of the KIs suggested that government must demonstrate willingness to be accountable in order to encourage private sector investment in public health programmes.

(See Quotes 1.6.1–1.6.5)

#### Public-private partnership

Respondents admitted that government can leverage private sector competencies in addressing certain challenges (R-1; R-3; R-4; R-5). As mentioned earlier, the private sector as well as wealthy individuals was cited as a potential source of financing for Nigeria’s HIV response (R-2; R-4; R-8; R-10; R-12) even though one of the respondents was of the opinion that with proper management and accountability systems, resources available within government should be sufficient to fund HIV program (R-3). The private sector can specifically be leveraged for capacity building and commodity distribution (R-8; R-12). One respondent remarked that government should “move away” from running the supply chain and another noted that donors may be more comfortable working through the private sector as they are more transparent and accountable (R-10; R-12).

(See Quotes 1.7.1–1.7.6.1).

#### Prevention

According to one of the respondents, government should prioritize prevention activities as this will help eliminate new infections (R-6) (See Quote 1.8.1).

### Healthcare financing

Healthcare financing ensures that there is adequate fund which should be equitably distributed for healthcare delivery. The vulnerable and the poor in the society must be protected from non-utilization of health services and impoverishment that may arise from having to pay for services [20].

#### Domestic funding

Respondents noted the country can be said to own its HIV response when it funds a large percentage of its response and locally manufactures HIV commodities (R-6; R-7; R-8; R-10). Some of the respondents remarked that Nigeria demonstrated willingness to increase local funding for HIV response through Presidents Comprehensive Response Plan (PCRP) which was predominantly funded through Subsidy Re-investment and Empowerment Programme (SURE-P). SURE-P funds are however not available any more but it appears government has plans to increase funds for the country’s HIV response through budgetary allocations (R-4; R-9; R-11; R-12). One of the respondents noted that PCRP has an element of fund mobilization from the private sector though it was not clear if this plan was implemented (R-11). Apart from national funding, respondents mentioned that some states now have budget line for logistics activities especially for the support of LMCU activities (R-6; R-7; R-8; R-11). In addition, one of the stakeholders at the national level mentioned that government has visibility into funding commitment by donors and government only bridges identified gaps (R-9).

According to respondents, inadequate domestic funding appears to be the biggest challenge hampering CO and that with adequate fund allocation, most of the other challenges can be effectively tackled (R-1; R-2; R-3; R-5; R-6; R-7; R-11; R-12). With the poor economic situation of the country, respondents stated that the challenge of inadequate funding may not be resolved anytime soon (R-2; R-5; R-7; R-10). One of the respondents suggested that donors have started withdrawing funding for certain activities such as HIV outreach testing (R-3). While some respondents mentioned that out-of-pocket payment by HIV patients may not be a viable option for funding Nigeria’s HIV response due to the poor economic status of HIV positive clients (R-2; R-4; R-5), some of them mentioned that the country could consider introduction of subsidized fees for HIV services as a means of engendering sustainability and improving adherence (R-3; R-6). Some respondents noted that inadequate resources may hinder implementation of test and treat policy (R-3; R-6). (See Quotes 2.1.1–2.1.10).

#### Bureaucratic bottlenecks

Bureaucratic bottlenecks came up as a challenge hampering CO. Respondents mentioned that funds budgeted by government are sometimes not released and when released it is either not timely or not in full; and in some cases, both (R-2; R-3; R-9; R-12). (See Quotes 2.2.1–2.2.2).

#### Alternative funding sources

A number of alternative funding sources were proffered. These include: private companies-though it was noted by some respondents that they should not be compelled by law to fund HIV response (as the poor economic situation is also negatively affecting their operations) but rather should be incentivized to do so through tax rebates (R-3; R-5; R-6; R-12), use of certain percentage of accrued profit from drug revolving fund (which one of the states of the federation is already implementing) (R-7) and expansion of NHIS coverage to the informal sector of the economy (R-3, R-7; R-9). High level advocacy to the relevant state stakeholders was also suggested as a means of catalyzing sub-national funding allocation (R-1).

In addition, response from one of the respondents suggests that government funding should extend beyond procurement but must also cover other supply chain expenses such as port clearance, distribution etc. (R-4). Finally, one of the respondents suggested that significant co-funding by government can eliminate “dictatorial tendency” of donors (R-11).

See Quotes (2.3.1–2.3.6).

### Health workforce

The health workforce refers to all the people responsible for healthcare service delivery. These individuals must be adequate, competent and well-motivated, equitably distributed and alive to their responsibilities [20].

#### Human resource for health (HRH), training and mentoring, capabilities and Behavioural disposition

Respondents remarked that virtually all the supply chain workforce at the lower levels are employed by the Nigerian Government (R-2; R-12) and that donor intervention has contributed significantly to capacity building across all levels (R-1; R-2; R-3; R-4; R-5; R-7; R-8; R-9; R-11; R-12). Some of the respondents confirmed the institutionalization of pre-service training in the schools of Pharmacy, Laboratory Sciences and Health Technology as a means of addressing supply chain competencies gap (R1; R-9).

While it appears that there is adequate capacity at the national level especially within the private sector (R-6; R-10; R12), there is dearth of manpower at the sub-national level especially at the health facilities; this situation is further compounded by Nigeria’s current poor economic situation which may not allow for personnel recruitment in the immediate future (R-1; R-2; R-3; R-4; R-5; R-7; R-12). Attrition/transfer of trained staff (R-1; R-2; R-5; R-7) was mentioned as a key human resource challenge. Remuneration differential was mentioned as one of the contributory factors to attrition with resultant inequitable distribution of healthcare professionals in some instances (R-7). Two respondents noted that there are still capacity gaps at the national and state levels especially for programme management and quantification of HIV/AIDS products(R-2; R-4). Another challenge mentioned by respondents is the poor work attitude and placement of personal gains above public good at the national level (R-2; R-8; R-11; R-12).

Recommendations suggested to address the human resource challenges include: advocacy to relevant authorities to reduce transfer of trained staff to sites where there skills may not be needed (R-1), recruitment, training and motivation of workers through regular payment of salaries and absorption of ad hoc staff under appropriate terms of service (R-2; R-3; R-5; R-7; R-11), prioritizing certain professionals for some SCM roles such as use of pharmacists as the preferred professional for pharmaceutical product selection (R-3), professional recognition of logisticians and better remuneration package for those performing SCM functions (R-3; R-4), task shifting (R-6), proper planning and regular training and mentorship for government officials as some changes are dependent on them (R-9; 12), institutionalizing and enforcing clear performance benchmarks to address attitudinal challenges (R-12), attracting best brains from the private sector to improve public sector performance (R-12) and institutionalizing health SCM as a professional postgraduate programme (R-3).

(See Quotes 3.1 .1–3.1.17).

### Medical products, technologies

This building block addresses the need to have a system that ensures equitable access to essential medicines, vaccines and technologies that have been scientifically proven to be cost-effective, safe and of good quality [20].

#### Local manufacturing

Local production of ARVs was mentioned as a potential metric for CO (R-6) (See Quote 4.1.1); this could foster price reduction and ensure continuous product availability (R-3; R-7; R-8) (See Quotes 4.1.2.0 and 4.1.2.1).

#### Unification and integration

Response from one of the KIs confirmed that Nigeria has a Unified HIV/AIDS Supply Chain System (R-8) which leveraged on existing in-country structures. KIs also noted that Nigeria is in the process of integrating supply chain systems across multiple programme areas through a project called Nigeria Supply Chain Integration Project (NSCIP) and that this project can benefit from the lessons learned from the HIV/AIDS Supply Chain Unification Project (R-1; R-2; R-8; R-10; R-12). One of the respondents noted that the LMCU will play significant role in this project especially as it relates to management and use of data (R-1). Two of the respondents think NSCIP will reduce the reporting burden on the healthcare workers through integration of reporting tools (R-1; R-2).

(See Quotes 1.4.3 and 4.2.1–4.2.4).

### Information and research

Information and research helps in ascertaining the overall health status of individuals and how good the system is. In a well-functioning system, information is expected to be timely and should be used in improving service delivery [20].

#### Data availability, data quality and data for action

LMCU was adjudged to have increased the use of data for decision making and visibility into happenings in other program areas (R-7), Respondents mentioned that logistics data is available and now of improved quality even though there may be need to put appropriate infrastructures in place for data management (R-1; R-2; R-4). Instances where data were used for advocacy and decision making (conduct of inter-facility redistribution) were cited (R-2; R-5; R-6; R-7; R-12). The LMCU was particularly mentioned as the unit responsible for data management and some respondents mentioned that the unit is developing quarterly stock status reports. The activity of the unit is also said to be improving the data quality for other program areas (R-2). Two respondents noted that the quality of data in other programme areas needs improvement (R-3; R-8).

Electronic data collection was suggested as a means of improving data quality (R-3; R-11).

(See Quotes 5.1.1–5.1.6).

### Service delivery

Service delivery can be adjudged to be good when it delivers health services (that are effective, safe, of good quality) in a timely fashion to individuals in need at the lowest cost possible [20].

#### Physical infrastructure

One of the major contributions of Nigerian government towards CO is making health facilities available for service delivery (see Quote 1.1.4). Nigeria has warehouses that can be used for commodity storage while donors have invested significantly in making appropriate storage facilities available. There was specific reference to Warehouse-in-a-Box (WiB) being set up in Abuja and Lagos States (R-2; R-4; R-5; R-6; R-8). One of the respondent mentioned that the country is embracing public-private Partnership in the management of WiB (R-9). There is also evidence of infrastructural donations of computers, chairs and tables to the LMCU (R-2). Respondents however decried the lack of vehicles for supportive supervision and the poor road network to some areas in the country (R-1; R-2; R-6).

(See Quotes 6.1.1–6.1.4).

#### Access

Response from a respondent suggests that new health facilities are being activated to improve access; more is however required to improve coverage (R-5; R- 6). One of the respondents noted that stigma has reduced and treatment acceptance has improved (R-8) while private HFs are being used to improve access (R-2; R-8) (See Quotes 6.2.1–6.2.4).

#### Integration

One key informant remarked that integration of HIV services into general outpatient care can help in reducing stigmatization (R-3). (see Quote 4.2.4).

## Discussions

### Understanding

Overall, respondents had a good understanding of CO and sustainability. KIs underscored the critical role of government leadership in engendering CO as well as the need for an all-inclusive stakeholders’ participation as indicated in the UNAIDS document. This finding is consistent with the report that strong political will was pivotal to the progress made by Botswana in fighting HIV/AIDS pandemic [15]. Some functions may also be contracted to the private sector [42]. The possibility of government directly funding non-governmental organizations (NGOs) as done in Guyana and Lesotho should also be explored [6]. Capability of local NGOs is being strengthened in this regard as PEPFAR has transitioned all Track 1 grants to local NGOs [20, 43].

KIs rightly noted that for the program to be sustainable, it must continue after the termination of donors’ support although some program modifications may be necessary [44, 45].

### Achievements

Strategic plans and policy frameworks are crucial for the attainment of CO and the country has made some progress in this regard as highlighted in the results section. It is however worrisome that the National Strategic Health Development Plan (2016–2020) which is expected to provide overarching policy guidance for the health sector is yet to be published; this may create some gap in concrete program direction [46]. The HIV-specific strategic plan (National HIV/AIDS Strategic Framework 2017–2021) has however been developed [11].

Key informants mentioned that Nigeria has made substantial progress with respect to coordination of the HIV/AIDS SCM program [33]. Findings from the interviews conducted by Chima and Homedes (2015) however suggested that Nigeria has not done a good job with the coordination of donor-funded programs [22]; while the researchers looked at the impact of global health initiatives (GHIs) on the overall HIV program, this study is only focused on SCM. They also reported that there may be some overlap in the coordination roles of NACA and NASCP, none of the respondents in this study however mentioned this [22].

The fact that LMCUs have representation from diverse public health programmes and have increased data availability and use (partly through the generation of quarterly stock status reports) is commendable. These findings are consistent with what Attah et al. (2015) reported in their abstract [34]. Instrumental to the successes recorded by the LMCUs is the support (both technical and infrastructural) provided by partners [34]. Inauguration of LMCUs across all the local government areas in the country was completed in November 2017 although operationalization is still at the preliminary stage. The country therefore needs to strengthen these units since primary health centres are under their direct supervision.

With improved availability of HIV/AIDS logistics data in the country, data is now being put into diverse uses such as: advocacy, identification of funding gap and correction of stock imbalance. This is a notable achievement as this will ensure that the right decisions which would have otherwise being impossible without quality data are made. Also noteworthy is that the LMCU structure has made it possible for the HIV programme to stimulate improvement in the quality of data for other programme areas.

Unification of Nigeria’s HIV/AIDS supply chain systems is a major achievement for the country and it was specifically acknowledged that existing government structures were leveraged for warehousing. Unification has also reduced stock outs – Aguora et al. (2014) reported a decline in stock outs from 25% to < 6% in the course of project implementation [22, 47]. As opposed to the report by Chima and Homedes (2015) that the system is not sustainable, Itiola et al. (2014) noted that the use of government warehouses and the now unitary nature of the supply chain will make it easier for government to operate the SCS if donor support is withdrawn [37]. While the claim that the supply chain is mainly run by a consortium of foreign organizations is correct, almost all the in-country staff are Nigerians which thus provides a pool of in-country supply chain experts. The Unification Project has also expanded in-country third party logistics companies market which thus places the private organizations in a good position to support logistics activities [35]. The success of Nigeria’s HIV/AIDS Supply Chain Unification Project is said to be influencing the approach being adopted for the ongoing Nigeria Supply Chain Integration Project.

As reported in diverse publications [10, 24, 36], Nigerian government set up PCRP in a bid to increase domestic financing even though the fund is no longer in operation there is evidence that the government is willing to increase domestic allocation. Domestic financing for HIV programme has grown over the years (from 7% in 2008 to 23% in 2012) but still falls below the agreed 50% benchmark in the partnership framework agreement PFA [15, 37]; budgetary allocation to the health sector is also below the minimum of 15% contained in the Abuja Declaration [38]. Sub-national funding of HIV/AIDS SCM (especially for LMCU activities) is however commendable as this will go a long way in augmenting federal allocation [39].

Government-paid staff in public health facilities (HFs) are mainly responsible for service delivery while new HFs are typically activated to improve access. Some individuals however prefer to access services from the private HFs. Government of Nigeria has also taken over provision of HIV treatment, care and support services in Taraba and Abia States (both states have ~ 35,000 patients on treatment) with primary funding from PCRP [24]. With the termination of PCRP fund, it appears the government plans to continue funding these states through direct budgetary allocation. This is a sign that Nigeria may be on her way towards CO as the country is taking up more responsibility for her HIV response.

Donor–funded programmes has built the capacity of Nigerians on SCM through in-service and pre-service trainings. The pre-service training which is adjudged to be more cost-effective and sustainable is expected to equip undergraduates with SCM management skills that will enable them perform SCM duties as soon as they graduate [40].

### Challenges

The biggest challenge mentioned by respondents is inadequate domestic financing which in turn has negatively affected implementation of other activities such as employment of adequate skilled staff and funding of LMCU activities. Inadequate financing is said to be compounded by the country’s poor economic condition due to dwindling oil prices as of the time interviews were completed (negative economic growth of −1.5% in 2016) [48]. Botswana was however able to fund substantial part of her HIV response despite dwindling resources from diamond export [15]. Nigeria’s precarious situation is largely due to the mono nature of her economy hence the government must put measures in place to diversify the economy. Recent data shows that Nigeria’s economic outlook has improved even though the growth rate of 0.8% in 2017 still falls below the average growth of 5.7% between 2006 and 2016 [48, 49]. Bureaucracy is one of the factors impeding CO as funds allocated are either not or partially released. There were also concerns around non-timeliness of releases.

Findings from this study revealed that the political class may not be showing enough commitment towards donor funded programme. Also noted is the lack of accountability and transparency within government and poor adherence to relevant procurement processes which sometimes result in the embezzlement of donor funding as seen in the case of Nigeria’s DPRS [41]. It is therefore important for transparent and strong financial management processes to be put in place and enforced in order for the government to regain the confidence of donors and the private sector.

Human resource challenge still persists, while there may be adequate capacity in the private sector, available human resource in the public sector is inadequate and do not have requisite SCM skills and competencies. Attrition of trained staff was also highlighted as a challenge-sometimes due to the quest for better pay packages either within the public sector or in private organizations. This is said to have also resulted in maldistribution of healthcare workers with the rural areas largely ill-staffed [22, 50]. In general, Africa is plagued with inadequate human resource for health and the developed countries have contributed to this shortage as Africa’s best brains migrate to these countries in search of greener pastures [51–53]. The number of pharmacists per 10,000 population in sub-Saharan Africa countries is less than one (Ethiopia has about 2.2 pharmacists per 100,000 population) while high income countries have the highest pharmacist to population ratio [29, 54]. This dearth of pharmacists in developing countries is said to have contributed to the poor access to medicines [29, 54].

Another challenge mentioned is poor attitude of government workforce and nepotism as personal gains are sometimes placed ahead of public good which ultimately impacts negatively on programme implementation. Lastly, a challenge which the country should prepare for is withdrawal of donor funding as funding for certain activities have been withdrawn [38] hence the country should be well prepared as it was reported that cessation of World Bank loan to Guyana led to termination of HIV funding for prevention services and to NGOs, South Africa lost 50,000 to 200,000 patients from care while there was rise in HIV prevalence among people who inject drugs in Romania Serbia and Belarus [6, 19, 25].

### Recommendations

The recommendations proffered in this study were mainly to address the human resource and inadequate domestic funding challenges.

Recommendations targeted at addressing human resource challenge include: advocacy to relevant stakeholders to reduce transfer of trained staff, motivation of workers through prompt payment of salaries and absorption of ad hoc staff under appropriate terms of service. Task shifting was also suggested as this has been a proven and effective method of addressing human resource challenges. Respondents also suggested that government should attract the best brains from the private sector which may be similar to what was done in Namibia where staff of Global Fund and PEPFAR were moved in a phased manner to government’s payroll [55]. To address attitudinal issues, respondents suggested that clear performance benchmarks should be set and enforced. The private sector can also be used for project implementation and management just as it is currently being planned for the management of Warehouse-in- a- Box (WiB).

Respondents also advocated for prioritization of some professionals for certain SCM tasks and special pay for those involved in SCM activities given the critical and tasking nature of their role. There was also a call for professionalization of SCM of heath commodities. As reported by O’Neil (2008), while millions of dollars are being invested in drug procurement, very little investment is going into making sustainable workforce available for service delivery [51]. Donors therefore need to seriously consider investing substantially in human resource for health although it may take some time for these investments to start yielding dividends [51].

In order to increase domestic funding for HIV/AIDS SCM, respondents suggested sourcing funds from the private sector through the use of incentivizes rather than legislation and expansion of NHIS coverage. Health insurance coverage in Nigeria stands at a paltry 5% with majority of the individuals covered being those employed in the formal sector [56]; out-of-pocket expenditure as a percentage of private healthcare expenditure is close to 96% in 2012. These statistics buttress the need to expand NHIS coverage especially for the informal sector. DRF is also a potential of HIV/AIDS SCM funding. This is currently ongoing in Jigawa and Kaduna States [15, 39] and other states can also explore this. High level advocacy is also suggested as an approach for catalyzing sub national funding. Evidence shows that some African countries like Botswana, Gabon, Namibia and South Africa that were previously donor-dependent now fund significant proportion of their HIV response [57, 58]. This suggests that with the right political will, Nigeria can achieve a similar status.

Respondents identified the need for infrastructural upgrade as this is critical in accessing some health facilities for supportive supervision which in turn will also affect service/product delivery.

The need to put favorable policies in place to enhance local production was particularly stressed. Most of the HIV commodities used in Nigeria are imported and this also extends to other health commodities. Strengthening local production will not only engender self-sufficiency but also create employment opportunities for Nigerians and ultimately boost the economy [15, 59].

Nigeria needs to maximize donors’ support by investing cost savings accruing from donor’s investment in other parts of the healthcare system. Government should also delibrately integrate best practices from donor-funded programs into the general healthcare system.

As acknowledged by KIs, donors exit should not be abrupt. Donors should work with host governments to develop and see to the implementation of a realistic exit plan that takes into account the prevailing socioeconomic conditions as recommended by UNAIDS [16]. The consideration should go beyond using gross national income per capita as the recent extension of Gavi graduation timeline for Nigeria from 2021 to 2028 suggests that this metric may be too simplistic [60].

### Existing opportunities

A number of opportunities some of which have been discussed under achievements were reported in this study. Notable among these are the Nigeria Supply Chain Integration Project, the NHIS, the private sector and academic institutions. Key informants felt integration and in particular data collection tools integration will reduce cost and reporting burden on healthcare workers. Lastly it appears that the potential of the academic research institutes in generating useful research findings that can help improve the SCS remains largely untapped. While there is evidence that academic institutions are now convening supply chain courses and conferences, more needs to be done in the area of generating transformative research findings.

Our study has a number of implications for other donor-supported countries; firstly, securing political buy-in is critical for attainment of CO and sustainability, secondly, countries must have a working definition for CO and sustainability as this will largely influence the approach for sustainability planning especially in the light of private sector involvement. Thirdly, CO and sustainability planning can start with the development of strategic plans and policies, however inadequate funding remains the biggest challenge that needs to be surmounted, hence, countries must be innovative in resource mobilization and should explore beyond budgetary allocations. This effort should be complemented with reduction in bureaucratic bottlenecks and strengthening of financial management processes. Lastly, human resource is a major driver for CO, and countries must work with donors to establish a workable human resource plan that will ensure availability of adequate and skilled HR to drive public health programmes post donor support.

### Limitations

Due to limited time available for this study, saturation was not pursued-we did not interview increasing number of respondents until new themes stop emerging. Also given that stakeholders had busy schedule, it was difficult to schedule a central meeting, focus group discussions (FGDs) were therefore not conducted. Manufacturers and HIV patients were also not included as respondents in this study hence the results presented did not capture their perception. This study was also limited to SCM component of HIV/AIDS programme. It is however anticipated that findings will also apply to other components of HIV/AIDS programme. Lastly, all results presented were based on the prevailing situation at the time the interviews were conducted and there may have been some changes as of now. Although our study is largely explorative, to the best our knowledge, no other publication in Nigeria has presented a SCM-specific review of CO and sustainability.

## Conclusion

Respondents demonstrated good understanding of CO and sustainability and acknowledged that Nigeria has made some progress especially in the area of developing strategic plans, having coordination structures, use of data for decision making, and unification of the hitherto parallel HIV/AIDS supply chains in the country. Human resource and funding challenges will however require effective healthcare workforce planning and mobilization of resources from alternative funding sources. Overall this study underscored the fact that HIV/AIDS SCS cannot be dissociated from the larger socioeconomic and political happenings and that for CO and sustainability to be attained, strong political commitment backed up with substantial financing are required. As other countries plan for CO and sustainability, it is important to secure political buy-in and adopt a working definition for CO and sustainability while resource mobilization and workforce planning should be prioritized.

## Additional files

Additional file 1: Nigeria’s HIV/AIDS Supply Chain System (SCS). (DOCX 86 kb)

Additional file 2: Overview of Nigeria’s Healthcare System. (DOCX 16 kb)

Additional file 3: Interview Guide. (DOCX 15 kb)

Additional file 4: List of Selected Quotes. (DOCX 30 kb)

Additional file 5: Ethics Approval. (DOCX 223 kb)

## Abbreviations

AIDS: Acquired immunodeficiency syndrome
ARVs: Antiretroviral drugs
CO: Country ownership
DPRS: Department of Planning Research and Statistics
DRF: Drug revolving fund
FGDs: Focus group discussions
GHIs: Global Health Initiatives
HF: Health facility
HIV: Human immunodeficiency virus
HR: Human resource
KIs: Key informants
LACA: Local Government Agency for the Control of AIDS
LG: Local government
LGA: Local government area
LMCU: Logistics Management Coordinating Unit
NACA: National Agency for the Control Of AIDS
NASCP: National AIDS and STI Control Program
NHIS: National Health Insurance Scheme
NPSCMP: National Product and Supply Chain Management Programme
NSCIP: Nigeria Supply Chain Integration Project
PCRP: President’s comprehensive response plan
PEPFAR: President’s Emergency Fund for AIDS Relief
PFA: Partnership framework agreement
PSM TWG: Procurement and Supply Management Technical Working Group
SACA: State Agency for the Control of AIDS
SCM: Supply chain management
SCS: Supply chain System
SCMS: Supply chain management system
STI: Sexually transmitted infection
SURE-P: Subsidy Re-Investment and Empowerment Programme
UNAIDS: Joint United Nations Programme on HIV/AIDS
WHO: World Health Organization
WiB: Warehouse –in- a- Box

## References

[1] WHO. HIV/AIDS. 2016. www.who.int/gho/hiv/en/ Accessed 31 Mar 2016.

[2] UNAIDS. The Gap Report. 2014. http://www.unaids.org/sites/default/files/media_asset/UNAIDS_Gap_report_en.pdf Accessed 21 July 2016.

[3] Khakshour A, Taghizadeh Moghadam H, Kiani MA, Saeidi M (2014). Key facts about epidemiology of HIV/AIDS in children worldwide. Int J Pediatr.

[4] The World Bank Group. Children (0–14) living with HIV | data | table. 2015. http://data.worldbank.org/indicator/SH.HIV.0014 Accessed 22 Feb 2015.

[5] UNAIDS.Global report: UNAIDS report on the global AIDS epidemic 2013. 2013. http://www.unaids.org/sites/default/files/media_asset/UNAIDS_Global_Report_2013_en_1.pdf Accessed 14 Feb 2015.

[6] Katz I, Glandon D, Wong W, Kargbo B, Ombam R, Singh S, Ramsammy L, Tal-Dia A, Seck I, Osika JS. Lessons learned from stakeholder-driven sustainability analysis of six national HIV Programmes. Health Policy Plan. 2014;29(3):379–87. 10.1093/heapol/czt024 23612848

[7] WHO. Number of people (all ages) living with HIV. 2016. http://www.who.int/gho/hiv/epidemic_status/cases_all_text/en/ Accessed 31 Mar 2016.

[8] Gona CM, McGee E, DeMarco R (2016). “What Will Become of Me if They Take This Away?” Zimbabwean Women’s Perceptions of “Free” ART. J Assoc Nurses AIDS Care.

[9] Bashorun A, Nguku P, Kawu I, Ngige E, Ogundiran A, Sabitu K, Nasidi A, Nsubuga P. A description of HIV prevalence trends in Nigeria from 2001 to 2010: What is the progress, where is the problem? Pan Afr Med J. 2014;18(1):3. 10.11694/pamj.supp.2014.18.1.4608 PMC4199354 25328622

[10] National Agency for the Control of AIDS. Federal Republic of Nigeria: Global AIDS Response Country Progress Report. 2014. http://www.unaids.org/sites/default/files/country/documents/NGA_narrative_report_2014.pdf. Accessed 27 July 2016

[11] National Agency for the Control of AIDS. National HIV and AIDS Strategic Plan 2017–2021. 2017. https://naca.gov.ng/wp-content/uploads/2018/05/National-HIV-and-AIDS-Strategic-Plan-FINAL1.pdf. Accessed 24 Apr 2018

[12] UNAIDS. Country: Seattle. 2018. https://digital.lib.washington.edu/researchworks/bitstream/handle/1773/23432/Ndoh_washington_0250O_12108.pdf?sequence=1. Accessed 4 Jan 2018

[13] Ibegbunam I, McGill D (2012). Health commodities management system: priorities and challenges. J Humanitarian Logist Supply Chain Manag.

[14] Ezegbe C, Stephenson N (2012). The reach and limits of the US president's emergency plan for aids relief (PEPFAR) funding of prevention of mother-to-child transmission (PMTCT) of HIV in Nigeria. Afr J Reprod Health.

[15] Ndoh KI. Funding sustainability for HIV/AIDS prevention, treatment and Care in Nigeria, master of public health degree. Seattle: Thesis submitted to University of Washington; 2013. https://digital.lib.washington.edu/researchworks/bitstream/handle/1773/23432/Ndoh_washington_0250O_12108.pdf?sequence=1.

[16] UNAIDS. Country Ownership for a Sustainable AIDS Response: From Principles to Practice. 2012. http://www.unaids.org/sites/default/files/sub_landing/files/20120717_JC2134_UNAIDS_Country_Ownership_Discussion_Paper.pdf Accessed 27 July 2016.

[17] National Academy of Sciences. Evaluation of PEPFAR 2013 Chapter: 10 progress toward transitioning to a sustainable response in partner countries. 2013 http://www.nap.edu/read/18256/chapter/23#545 Accessed 09 Aug 2016.

[18] Lantos T, United Hyde HJ. States Global Leadership Against HIV/AIDS, Tuberculosis, and Malaria Reauthorization Act of 2008. Public law. 2008;110:293 http://www.pepfar.gov/documents/organization/108294.pdf Accessed 10 Aug 2016.

[19] Vogus A, Graff K. PEPFAR Transitions to Country Ownership: Review of Past Donor Transitions and Application of Lessons Learned to the Eastern Caribbean. Global Health: Science and Practice. 2015; 3(2): 274–286. http://www.ncbi.nlm.nih.gov/pmc/articles/PMC4476864/. Accessed 21 July 2016. 10.9745/GHSP-D-14-00227 PMC4476864 26085023

[20] USG. U.S. Government Interagency Paper on Country Ownership - Global Health Initiative. 2012. https://pdf.usaid.gov/pdf_docs/pnaec184.pdf. Downloaded 10 Apr 2015.

[21] WHO. The WHO Health Systems Framework. 2018. http://www.wpro.who.int/health_services/health_systems_framework/en/. Accessed 4 Jan 2018.

[22] Chima CC, Homedes N. Impact of Global Health Governance on Country Health Systems: HIV Initiatives in Nigeria. Journal of Global Health. 2015; 5(1):010407 Available: https://www.ncbi.nlm.nih.gov/pmc/articles/PMC4416331/ Accessed 01 June 16. 10.7189/jogh.05.010407 PMC4416331 25969731

[23] Moszynski P. Donor fatigue is slashing access to AIDS care in Africa, warns charity, BMJ. 2010;340:c2844. http://www.bmj.com/content/340/bmj.c2844. Accessed 06 Aug 2016. 10.1136/bmj.c2844 20508020

[24] Olakunde BO, Ndukwe CD. Improved Domestic Funding Enhances the Sustainability of HIV/AIDS Response in Nigeria. Ann Glob Health. 2015;81(5) 684–688 https://www.sciencedirect.com/science/article/pii/S2214999615012655?via%3Dihub. Accessed 06 Aug 2016. 10.1016/j.aogh.2015.10.005 27036726

[25] Burrows D, Oberth G, Parsons D, McCallum L. Transitions from donor funding to domestic reliance for HIV responses Recommendations for transitioning countries. 2016. http://www.globalfundadvocatesnetwork.org/wp-content/uploads/2016/04/Aidspan-APMG-2016-Transition-from-Donor-Funding.pdf. Accessed 6 Apr 2018.

[26] Management Sciences for Health. Health Systems in Action: An e- Handbook for Leaders and Managers. Cambridge, MA: Management Sciences for Health. 2010. http://www.msh.org/sites/msh.org/files/ehandbook_2014_final_29aug14.pdf. Accessed 20 Dec 2011.

[27] USAID | DELIVER PROJECT. The Logistics Handbook: A Practical Guide for the Supply Chain Management of Health Commodities. Arlington, Va.: USAID | DELIVER PROJECT, Task Order 1. 2011.

[28] Durokifa A, Abdul-Wasi M. Evaluating Nigeria’s Achievement of the Millennium Development Goals (MDGs): determinants, deliverable and shortfalls. Africa’s Public Service Delivery and Performance Review. 2016; 4(4):656–683 https://journals.co.za/content/journal/10520/EJC-57a97935a. Accessed 1 Jan 2018.

[29] Kälvemark SS, Traulsen JM, Damene KW, Mekasha HB, Teshome GD, Essah NA, Khan SA, Brown AN. Developing and sustaining human resources in the health supply chain in Ethiopia: barriers and enablers. Rural and Remote Health. 2016; 16: 3613. http://www.rrh.org.au/publishedarticles/article_print_3613.pdf. Accessed 10 Aug 2016 27487268

[30] Yadav P (2015). Health product supply chains in developing countries: diagnosis of the root causes of underperformance and an agenda for reform. J Health Syst Reform.

[31] Federal Ministry of Health (2016). National Guidelines for HIV Prevention Treatment and Care.

[32] WHO. Supply and Management of Commodities. 2016. http://www.who.int/hiv/topics/vct/toolkit/components/supply/en/. Accessed 07 Aug 2016.

[33] Ibegbunam I, Aguora S, Raji J, Adedoyin A, Yekini O, Otohabru B, Odelola B. Donor Coordination Through Nigeria HIV/AIDS Procurement and Supply Management: Platform to Maximize Use of Limited Resources and Strengthen National System for Sustainability. 2014. (abstract accepted for poster presentation at International AIDS Conference held in July 2014 in Melbourne Australia).

[34] Attah M, Ibegbunam I, Efem I, Adedoyin A, Odelola B, Mohammed A, Chiazor E, Aguora S, Durosinmi-Etti O, Nwuba C, Haruna J, Iwheye-Adie B, Bonatson J, James D, Stephen A (2015). A Coordinated Approach to Promote State Government Visibility and Ownership of Public Health Supply Chains in Nigeria: The State Logistics Management Coordinating Unit (LMCU) Model.

[35] Itiola A, Obi C, Raji J, Ibeme I, Ibegbunam I, Aguora S, Olalandu W (2014). Government Partnership and Private Sector Engagement: Drivers for optimal performance of Nigeria HIV/AIDS Supply Chain System.

[36] Etsetowaghan A, Adenusi P, Emidio O, Umoru I, Akinseye M, Adebiyi O, Ugwoeruchukwu W. The Presidential Comprehensive Response Plan for HIV/AIDS in Nigeria 2013–2015: A 6-Month Review. 2014. Abstract Accepted for Poster Presentation at IAS 2014 .http://pag.aids2014.org/EPosterHandler.axd?aid=469. Accessed 06 Aug 2016.

[37] The Government of Nigeria and The United States Government. The Government of Nigeria and The United States Government. Partnership Framework on HIV/AIDS 2010–2015. A memorandum of understanding between the Government of Nigeria and the United States Government to fight HIV/AIDS in Nigeria, 2010. 2010. https://ng.usembassy.gov/embassy-consulate/abuja/sections-offices/pepfar/. Accessed 06 Aug 2016.

[38] Organization of African Unity. African Summit on HIV/AIDS, Tuberculosis and Other Related Infectious Diseases Abuja, Nigeria 24–27 April 2001. 2001. http://www.un.org/ga/aids/pdf/abuja_declaration.pdf. Accessed 06 Aug 2016.

[39] Stephen A, Alawode S, Mohammed A, Attah M, Haruna J, Aguora S, Ibegbunam I, Dambo E, Amos G, Umar YA, Tockan SH, Bulus JM (2015). Stimulating government financing of public health supply chain functions in resource constrained settings: A case study of the Kaduna State Ministry of Health Supply Chain Management Coordinating Unit.

[40] Adekola A, Adelanwa A. Developing the SCM workforce in Nigeria through contextualised pre-service education and continued professional development. J Pharm Policy Pract. 2014;7(1). http://www.ncbi.nlm.nih.gov/pmc/articles/PMC4304315/#. Accessed 30 Aug 2016.

[41] The Global Fund Office of the Inspector General. Investigation Report Global Fund Grants to Nigeria Department of Health Planning, Research and Statistics. 2016. https://www.theglobalfund.org/media/2656/oig_gf-oig-16-015_report_en.pdf. Accessed 21 July 2016.

[42] Bornbusch A, Dickens T, Hart C, Wright C. A stewardship approach to shaping the future of public health supply chain systems. Global Health: Science and Practice. 2014; 2(4): 403–409. http://www.ncbi.nlm.nih.gov/pmc/articles/PMC4307857/. Accessed 10 Aug 2016. 10.9745/GHSP-D-14-00123 PMC4307857 25611475

[43] Catholic Relief Services. AIDSRelief Nigeria: Strengthening local health networks for sustainable HIV care and treatment. 2012. http://www.crs.org/sites/default/files/tools-research/aidsrelief-nigeria-strengthening-local-health-networks-hiv.pdf. Accessed 01 June 2016.

[44] Shediac-Rizkallah MC, Bone LR (1998). Planning for the sustainability of community-based health programs: conceptual frameworks and future directions for research, practice and policy. Health Educ Res.

[45] LaPelle NR, Zapka J, Ockene, J K. Sustainability of Public Health Programs: The Example of Tobacco Treatment Services in Massachusetts. Am J Public Health. 2006; 96(8): 1363–1369. http://www.ncbi.nlm.nih.gov/pmc/articles/PMC1522114/. Accessed 10 Apr 2016. 10.2105/AJPH.2005.067124 PMC1522114 16809592

[46] NACA. National HIV/AIDS Strategic Plan 2010–2015. 2010. http://www.ilo.org/wcmsp5/groups/public/%2D%2D-ed_protect/%2D%2D-protrav/%2D%2D-ilo_aids/documents/legaldocument/wcms_146389.pdf. Accessed 10 Aug 2016.

[47] Aguora S, Okafor E, Ibegbunam I, Adedoyin D, Iwheye-Adie B, Iyeme E, Otohabru B, Yekini O, Odelola B, Badiane K. HIV/AIDS supply chain unification: improving supply chain performance through coordination and efficient resource utilization. 2014. (Abstract accepted for poster presentation at International AIDS Conference held in July 2014 in Melbourne Australia). http://pag.aids2014.org/Abstracts.aspx?AID=5862. Accessed 25 Jun 2018.

[48] World Bank Group. The World Bank in Nigeria: Overview. 2018. http://www.worldbank.org/en/country/nigeria/overview. Accessed 21 Jun 2018.

[49] World Bank Group. Poverty & Equity Data Portal: Nigeria. 2018. http://povertydata.worldbank.org/poverty/country/NGA. Accessed 22 Jun 2018.

[50] Okpani AI, Abimbola S. Operationalizing universal health coverage in Nigeria through social health insurance. Nigerian Medical Journal. 2015; 56(5):305–310. http://www.ncbi.nlm.nih.gov/pmc/articles/PMC4698843/. Accessed 31 Aug 2016 10.4103/0300-1652.170382 PMC4698843 26778879

[51] O'Neil ML. Human resource leadership: the key to improved results in health. Human resources for health. 2008;6(1):1 https://human-resources-health.biomedcentral.com/articles/10.1186/1478-4491-6-10. Accessed 10 Aug 2016. 10.1186/1478-4491-6-10 PMC2440377 18570657

[52] Naicker S, Plange-Rhule J, Tutt RC, Eastwood JB. Shortage of healthcare workers in developing countries--Africa. Ethnicity and Disease. 2009; 19(1): 60. http://txfvzgw.ishib.org/journal/19-1s1/ethn-19-01s1-60.pdf. Accessed 10 Aug 2016. 19484878

[53] Waako PJ, Odoi-Adome R, Obua C, Owino E, Tumwikirize W, Ogwal-Okeng J, Anokbonggo, WW, Matowe L, Aupont. Existing capacity to manage pharmaceuticals and related commodities in East Africa: an assessment with specific reference to antiretroviral therapy. Hum Resour Health. 2009; 7(21). https://human-resources-health.biomedcentral.com/articles/10.1186/1478-4491-7-21. Accessed 21 Jul 2016. 10.1186/1478-4491-7-21 PMC2662782 19272134

[54] International Pharmaceutical Federation. FIP Global Pharmacy Workforce Report. 2012. http://www.fip.org/files/members/library/FIP_workforce_Report_2012.pdf. Accessed 10 Aug 2016.

[55] PEPFAR. PEPFAR supporting country ownership, key to a sustainable response. https://photos.state.gov/libraries/malawi/217630/PDFs/PEPFAR-Country%20Ownership.pdf.

[56] Awosusi, A., Folaranmi, T. and Yates, R. (2015). Nigeria's new government and public financing for universal health coverage. Lancet Glob Health 2015; (3)9:e514–e515 http://thelancet.com/journals/langlo/article/PIIS2214-109X(15)00088-1/fulltext. Accessed 31 Aug 2016. 10.1016/S2214-109X(15)00088-1 26275321

[57] Resch, S.,Ryckman, T. and Hecht, R. (2015). Funding AIDS programmes in the era of shared responsibility: an analysis of domestic spending in 12 low-income and middle-income countries. Lancet Glob Health, 3(1) e52 - e61 [Online] Available: http://thelancet.com/journals/langlo/article/PIIS2214-109X(14)70342-0/fulltext. Accessed 06 Aug 2016. 10.1016/S2214-109X(14)70342-0 25539970

[58] UNAIDS (2013b). Ten years since PEPFAR’s launch: the United States continues its leadership in the AIDS response. 2013. http://www.unaids.org/en/resources/presscentre/featurestories/2013/february/20130214pepfar10. Accessed 06 Aug 2016.

[59] Garuba HA, Kohler JC, Huisman AM (2009). Transparency in Nigeria's public pharmaceutical sector: perceptions from policy makers. Glob Health.

[60] Gavi. Gavi Board approves funding for inactivated poliovirus vaccine until 2020: Flexible support to Nigeria and post-transition countries also approved. 2018 https://www.gavi.org/library/news/press-releases/2018/gavi-board-approves-funding-for-inactivated-poliovirus-vaccine-until-2020/. Accessed 1 Jul 2018.

