# Supplementary material for: Country ownership and sustainability of Nigeria’s HIV/AIDS Supply Chain System: qualitative perceptions of progress, challenges and prospects
Source: J Pharm Policy Pract. 2018 Sep 10;11:21. doi: 10.1186/s40545-018-0148-8 (PMC6130083; doi:10.1186/s40545-018-0148-8)
Supplement: Supplementary file 1 — Nigeria’s HIV/AIDS Supply Chain System (SCS). (DOCX 86 kb) [file 40545_2018_148_MOESM1_ESM.docx]

**Nigeria’s HIV/AIDS Supply Chain System (SCMS)**


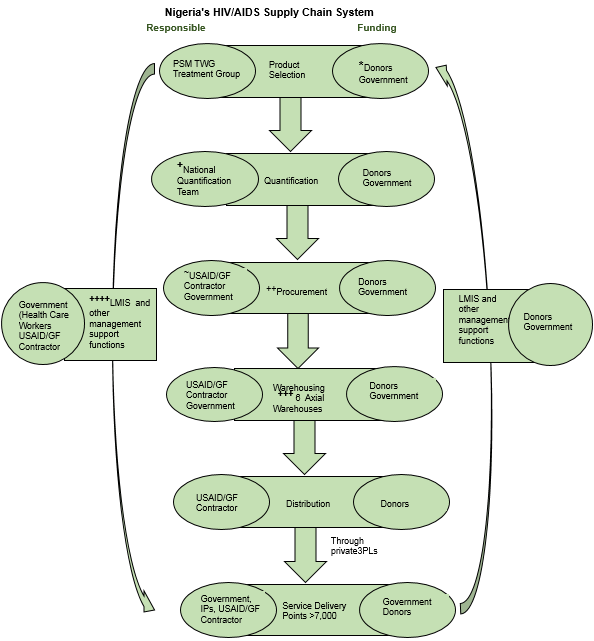


PSM TWG, Procurement and Supply Management Technical Working Group; USAID, United States Agency for International Development; GF, Global Fund; IPs, Implementing Partners; 3PL, Third Party Logistics; LMIS, Logistics Management Information System *Donors in Nigeria include Presidents Emergency Plan for AIDS Relief PEPFAR, Global Fund and World Bank; ^+^National Quantification Team works under the oversight of PSM TWG; ^~^Contractors involved in procurement include a consortium led by Partnership for Supply Chain Management (PFSCM) (during Supply Chain Management System (SCMS) project) and Chemonics International (with the current Global Health Supply Chain – Procurement and Supply Management project) ^++^HIV/AIDS Commodities are currently pool procured, ^+++^Distribution is carried out directly from the six axial (central) warehouses to over 7,000 service delivery points using 3PLs; ^++++^Health facilities submit LMIS reports in form of Combined Report Requisition Issue and Receipt Form (CRRIRF) report every two months and are resupplied to preset maximum at the same frequency

Nigeria’s HIV/AIDS SCMS was designed in 2005 and redesigned in 2008 in order to ensure that the system meets the challenges of ART services scale up^[[1]](#footnote-1)^. At the beginning of Nigeria’s HIV response, individual implementing partners operated their separate supply chain systems to procure and deliver health commodities to health facilities^[[2]](#footnote-2)^. This approach was adjudged to be expensive, prone to maldistribution of health commodities (with resultant wastage) and unsustainable^2^. To address these drawbacks, the unification of these parallel supply chain systems commenced in a phased manner in July 2012 and was completed in July 2014^2^. In the unified system, states in the country were clustered around six regional warehouses based on geographical proximity^2^. Commodities are delivered directly from these regional warehouses to health facilities on a bimonthly basis which makes the logistics structure a 2-tier system^2,^^[[3]](#footnote-3)^.

For inventory management, Nigeria operates a forced ordering maximum-minimum inventory control system. The implication of this system is that inventory levels must be kept within predetermined minimum and maximum stock levels while health facilities are expected to place orders at the end of the review period (2 months) to bring their stock level up to the preset maximum irrespective of whether or not they fall below the minimum stock level using the combined report requisition issue and receipt form (CRRIRF) (logistics report)^3,^^[[4]](#footnote-4)^. While the comprehensive antiretroviral therapy (ART) sites use the comprehensive CRRIRF which allow them to determine their resupply quantities, health facilities providing prevention of mother to child transmission of HIV (PMTCT) and HIV testing and counselling (HTC) services only use a simplified CRRIRF designed in July 2013 (the form was designed to address the issue of poor quality associated with the comprehensive form as staff at these HFs usually have lower educational qualification). Resupply quantities for PMTCT and HTC sites are determined by the staff working at the central level. Itiola *et al*. (2015) reported that the simplified CRRIRF improved the quality as well as reporting rate in Nigeria^[[5]](#footnote-5)^.

1. Ibegbunam I, McGill D. Health Commodities Management System: Priorities and Challenges. Journal of Humanitarian Logistics and Supply Chain Management. 2012; 2(2):161-182 [↑](#footnote-ref-1)
2. Itiola A, Obi C, Raji J, Ibeme I, Ibegbunam I, Aguora S, Olalandu W. Government Partnership and Private Sector Engagement: Drivers for optimal performance of Nigeria HIV/AIDS Supply Chain System. 2014. (accepted for oral presentation at 7th Global Health Supply Chain Summit held in Nov 2014 in Copenhagen Denmark) [↑](#footnote-ref-2)
3. Federal Ministry of Health. Logistics management of HIV/AIDS commodities, Standard Operating Procedures Manual for the Management of HIV/AIDS Commodities, Antiretroviral Drugs, OI Drugs, Laboratory Reagents and Supplies. 2011 [↑](#footnote-ref-3)
4. USAID | DELIVER PROJECT. The Logistics Handbook: A Practical Guide for the Supply Chain Management of Health Commodities. Arlington, Va.: USAID | DELIVER PROJECT, Task Order 1. 2011. [↑](#footnote-ref-4)
5. Itiola, A., Mohammed, A., Attah, M., Stephen, A., Haruna, J., Iwheye-Adie, B., Obi, C., Raji, J., Ibeme, A., Ibegbunam, A., Aguora, S., Yekini, O., Agabus, J., Echeta, T. and Otohabru, B. Simplification of logistics tools and mixed report collection models: Agents of HIV/AIDS commodity security in the era of rapid program scale up in Nigeria. 2015. (accepted for oral presentation at 8th Global Health Supply Chain Summit held in Nov 2015 in Dakar Senegal) [↑](#footnote-ref-5)
