# Supplementary material for: Country ownership and sustainability of Nigeria’s HIV/AIDS Supply Chain System: qualitative perceptions of progress, challenges and prospects
Source: J Pharm Policy Pract. 2018 Sep 10;11:21. doi: 10.1186/s40545-018-0148-8 (PMC6130083; doi:10.1186/s40545-018-0148-8)
Supplement: Supplementary file 2 — Overview of Nigeria’s Healthcare System. (DOCX 16 kb) [file 40545_2018_148_MOESM2_ESM.docx]

**Overview of Nigeria’s Demographics and Healthcare System**

Nigeria is a federal republic with three levels of government: federal, state and local government^[[1]](#footnote-1)^. There are 36 states and the FCT in the country which are further subdivided into 774 local government areas and 9,565 wards^1^. The population of Nigeria is estimated to be ~191 million as of 2017 with an estimated life expectancy of 55 years and 56 years for men and women respectively^[[2]](#footnote-2),^^[[3]](#footnote-3)^ The bulk (90% of export and 75% of consolidated budgetary revenues) of Nigeria’s revenue is from crude oil; accrued revenue is shared among the three levels of government^1,^^[[4]](#footnote-4)^. The country’s gross domestic product (GDP) is projected at 375.8 billion USD with total healthcare expenditure as percentage of GDP of 3.6% as of 2015^[[5]](#footnote-5),^^[[6]](#footnote-6)^. Approximately 54% of Nigerians live on <1.9 USD a day with a GNI per capita of 2,080 USD^2^.

The healthcare system operates through a 3-tier structure and there are three levels of care in Nigeria: Tertiary Secondary and Primary^1^. Tertiary care is coordinated by the federal government while secondary and primary care are under state and local governments respectively^1^. The federal government also provides overall policy guidance for health in Nigeria^1^. There are 23,640 health facilities in Nigeria as of 2005 close to 86% of which are primary health centers while 62% are publicly owned^1^ All levels of government are largely autonomous in allocating funds for healthcare. Sixty nine percent (69%) of the healthcare services in Nigeria are paid for out of pocket^1^.

The National Council on Health (NCH) is the highest health policy advisory body in Nigeria while the National Strategic Health Development Plan (NSHDP) provides the overall policy guidance for health^1^. The plan is developed through a consultative forum involving government stakeholders, partners and academia^1^. Decision on health interventions to be prioritized follows a systematic process and takes into consideration the disease burden in the country^1^. Progress is measured through various surveys such as National Nutrition and Health Survey (NNHS), National Demographic and Health Survey (NDHS) and Multiple Indicator Cluster Survey/National Immunization Coverage Survey (MICS/NICS)^1^. While there are couple of cost effectiveness studies for various health interventions in Nigeria, it is not clear whether these studies influence overall healthcare prioritization^[[7]](#footnote-7)^. The Nigeria Centre for Disease Control (NCDC) in Nigeria coordinates disease control and prevention efforts in the country^[[8]](#footnote-8)^. Efforts in the country targeted at preventing disease among other interventions include public education, vaccination and the use of chemoprophylactic agents^4,^^[[9]](#footnote-9)^.

1. Federal Ministry of Health. National Strategic Health Development Plan 2010 – 2015. 2010. [www.health.gov.ng/doc/NSHDP.pdf](http://www.health.gov.ng/doc/NSHDP.pdf). Accessed 10 Jul 2016 [↑](#footnote-ref-1)
2. The World Bank Group. Poverty & Equity Data Portal, Nigeria. 2018a <http://povertydata.worldbank.org/poverty/country/NGA> Accessed 10 June 2018 [↑](#footnote-ref-2)
3. WHO. Nigeria. 2018. <http://www.who.int/countries/nga/en/> Accessed 9 Jun 2018 [↑](#footnote-ref-3)
4. National Agency for the Control of AIDS. National HIV and AIDS Strategic Plan 2017- 2021. 2017. https://naca.gov.ng/wp-content/uploads/2018/05/National-HIV-and-AIDS-Strategic-Plan-FINAL1.pdf Accessed 24 Apr 2018 [↑](#footnote-ref-4)
5. The World Bank Group. Data Bank||World Development Indicators. 2018. <http://databank.worldbank.org/data/reports.aspx?source=2&country=NGA> Accessed 15 Jun 2018 [↑](#footnote-ref-5)
6. WHO. Global Health Expenditure Database. 2014 <http://apps.who.int/nha/database/ViewData/Indicators/en> Accessed 12 Jun 2018 [↑](#footnote-ref-6)
7. Wiseman V, Mitton C, Doyle‐Waters MM, Drake T, Conteh L, Newall AT, Onwujekwe O, Jan S. Using Economic Evidence to Set Healthcare Priorities in Low‐Income and Lower‐Middle‐Income Countries: A Systematic Review of Methodological Frameworks. Health Econ. 2016; 25:140–161. <https://onlinelibrary.wiley.com/doi/abs/10.1002/hec.3299> Accessed 28 Jun 2018 [↑](#footnote-ref-7)
8. NCDC. About NCDC. 2018. <https://ncdc.gov.ng/ncdc> Accessed 12 Jun 2018 [↑](#footnote-ref-8)
9. NPHCDA. What we do. 2018. <http://nphcda.gov.ng/about-us/what-we-do/> Accessed 11 Jun 2018 [↑](#footnote-ref-9)
