# Supplementary material for: Country ownership and sustainability of Nigeria’s HIV/AIDS Supply Chain System: qualitative perceptions of progress, challenges and prospects
Source: J Pharm Policy Pract. 2018 Sep 10;11:21. doi: 10.1186/s40545-018-0148-8 (PMC6130083; doi:10.1186/s40545-018-0148-8)
Supplement: Supplementary file 3 — Interview Guide. (DOCX 15 kb) [file 40545_2018_148_MOESM3_ESM.docx]

**Interview Guide**

|  | |
| --- | --- |
| - Could you please introduce yourself (including name, age or age range, sector of engagement (public or private), years of experience in HIV/AIDS commodity logistics) | |
| - Can you share what you understand by country ownership and sustainability? | |
| - In your opinion do you think Nigeria has made any progress with respect to country ownership and sustainability? | |
| - If yes, what progress has been made with respect to | |
|  | |
| - Political Ownership and Stewardship (Government commitment, availability of national plan) | |
|  | |
| - Institutional and Community Ownership (Public and private institutions to drive supply chain functions) | |
|  | |
| - Capabilities (Individuals with competencies in product selection, quantification, procurement, storage and distribution, Logistics Management Information System and Service Delivery) | |
|  | |
| - Mutual Accountability including finance (government/private sector financing of HIV programme | |
|  | |
| - What do you think are the challenges hampering country ownership and sustainability of HIV/AIDS logistics system in Nigeria? | |
| - How do you think Nigeria can overcome these challenges? | |
| - Are there further opportunities that the country can leverage on to engender country ownership and sustainability? | |
