# Supplementary material for: Country ownership and sustainability of Nigeria’s HIV/AIDS Supply Chain System: qualitative perceptions of progress, challenges and prospects
Source: J Pharm Policy Pract. 2018 Sep 10;11:21. doi: 10.1186/s40545-018-0148-8 (PMC6130083; doi:10.1186/s40545-018-0148-8)
Supplement: Supplementary file 4 — List of Selected Quotes. (DOCX 30 kb) [file 40545_2018_148_MOESM4_ESM.docx]

List of Selected Quotes

| **Code** | **Quote** |
| --- | --- |
| **1.0** | **Leadership and Governance** |
| **1.1** | **Government Leadership, Government Commitment, Political Will** |
| 1.1.1.0 | Ownership, I see ownership in the context of em…proprietorship in the sense that if an individual say[s] this thing belongs to me I own [it], you are definitely talking about proprietorship. but emm…when it comes to drug logistics or supply chain management ownership will definitely demand that very serious responsibility. So, if government says they own this program we then expect that government has taken very a large chunk of responsibility especially in the area of procurement, in the area of management of commodities and things like that. (R-4) |
| 1.1.1.1 | That is the easiest way to strengthen the system at least with the government being on the driver’s seat providing the strategic direction (R-11) |
| 1.1.2.0 | …CO doesn’t mean government must do everything; government just need to provide direction and ensure that people, donor, private sector align and when new information new knowledge new technology comes around government should be menable to align with best practices that the country can sustain rather than insisting this is the best way (R-12) |
| 1.1.2.1 | By that, we understand that the entire country has a government, then the people, the communities plus including everybody should be involved in the ownership it is not a one man’s ehm..job. I believe [CO] is an all-round pursuit: the government the community the people even the carriers themselves (R-2) |
| 1.1.3.0 | One thing I cannot predict, supply chain is evolving; it is very dynamic, it is not static. Today we have a system, tomorrow there may be need to modify the system so it is open...[it] is flexible and is open for modification when new technology or when new design comes up. (R-9) |
| 1.1.3.1 | this is my way it is either may way or [no other person’s] way because I am government sometimes your way may actually not be the best way but if you listen to reasoning and also adopt best practices and it must not be implemented by government then we are moving in the right path (R-12) |
| 1.1.4.0 | Nigeria has worked and has put in place all these things in support of partners that is what we should know. Then in the issue of provision of the facilities in themselves who will provide the services which I believe the government also is the one that is providing these facilities, there is also an issue of the staff that will manage the resources, which are also being paid by the government so in one way or the other (R-8) |
| 1.1.5.0 | But I think NSCIP now are using Taraba and Abia State to actually em…show, to pilot CO and em I think [they] also made available funds especially when there was em…SURE-P (R-2) |
| 1.1.6.0 | say number one is leadership, leadership and governance structure is a major challenge so that…..we can turn our policies into actual implementation that can actually be seen through in terms of implementation so leadership and governance is a major challenge (R-12) |
| 1.1.7.0 | There is probably, we might need a kind of powerful advocacy to federal government to see this ownership drive so that they will see [it] as their project not that somebody come to sit down in this area. (R-1) |
| **1.2** | **Continuity** |
| 1.2.1.0 | It means as a state or as a country we must the able to drive the process by ourselves, we must be able to own it and own it properly it therefore means in a situation…whereby donor support are withdrawn and we have our local arrangement to fund all the interventions (R-7) |
| 1.2.2.0 | It is the only way that the programme can be sustained in the long term. It is the only way but what they [the HIV clients] will be paying will be small and I have my reasons (R-3) |
| **1.3** | **Strategic Plan and Policies** |
| 1.3.1.0 | ..if you are going to have ownership there are certain things that are very very important which government actually have done. Number 1, policy framework. We have quality framework that…defined where government is going. We have things like emm Treatment Guideline because without treatment guideline, you cannot have a coordinated response against the disease (R-4) |
| 1.3.1.1 | ….You know, we have this National Strategic Plan NSP that speaks to it which is constantly being reviewed according to the existing government policy. That’s just the truth (R-9) |
| 1.3.2.0 | The program working with the partner has been able to develop at least the first National Pharmaceutical Supply Chain Policy it has been just signed off earlier in the year. Of course the policy still has little weakness because it is not yet clearly articulated because some things are still at the infant stage in the development stages of public health supply chain in Nigeria. I think there are plans currently underway to develop an implementation plan out of that policy that will now lead to the national plan. It is not yet there if it is there I am not aware anyway. It is not yet there but hopefully the output of that plan will … give a clear structure of how the program will move forward (R-12) |
| **1.4** | **Coordination and Coordinating Structures** |
| 1.4.1 | we could see so many things put in place to ensure that partners em. doesn’t just come and dump their work we want them to strengthen the system so that by the time the system, the system institution, people will be able to perform with minimal supervision….. The establishment of NASCP is there, (R-1) |
| 1.4.2 | this National [Product and] Supply Chain [Management Programme]…… at the national level, at state level we have PSM TWG, LMCU. All these things are to ensure that sustainability and ownership are really entrenched into our system (R-1) |
| 1.4.3 | All the programs, different different programs TB, malaria, HIV, neglected tropical diseases, nutrition, immunization, their logistics officers are part of their they are part of the LMCU (R-1) |
| 1.4.4 | The last meeting we had, we talked about having it [LMCU] in the Local government. You know we have [Primary] health centers under the Local Government while secondary and tertiary [are] under the state government. So they are thinking of extending it to the local government (R-5) |
| 1.4.5 | .. infact I made proposal to the state government and then we itemize what we need in that LMCU office, so the proposal is still on, so we hope that probably with time they will release some funds but actually it is probably about 2 months or so that I wrote, you know mostly bureaucracy with the state is very high so and then of recent they are also complaining of paucity of funds specifically in the past two months but then I’m definitely sure when the fund is available, they will make provision for that. (R-6) |
| **1.5** | **Donor Support** |
| 1.5.1.0 | .. Government ownership of the system you know has increased over the years em. Initially SC for HIV/AIDS was a purely donor driven affair and even though right now the donors are probably picking up about 95% of the cost government is more involved in coordination (R-10) |
| 1.5.1.1 | Well for now, we have dedicated six warehouses, each one for each geo-political zone through which the states in each zone have access to the commodities (R-8) |
| 1.5.2 | Our prayer is that em. the partners will continue to support us for a while (R-7) |
| 1.5.3 | so that when one idea improves the quality of health service delivery it rubs off positively also on the entire service delivery component (R-12) |
| 1.5.4 | …..it is expected that sometimes the donors will have to focus on some other interventions…. focus on some other countries that are less developed than Nigeria (R-7) |
| **1.6** | **Transparency and Accountability** |
| 1.6.1 | Nigeria Procurement Act, it is a very robust act it is a very robust system but the implementation of that act is the challenge…..…(R-12) |
| 1.6.1.1 | The thing is, you know in Nigeria now there is this Procurement Act, Public Procurement Act if implemented only to the letters you will not have any problem, you will not have any problem it is there it is just the implementation so the act is there I have read through it and discovered that minor things need to be amended and that is all that is all it is there but it is just the implementation that is the problem (R-11) |
| 1.6.2 | Okay in terms of transparency, you know we know Nigerians are....so the situation is the same anywhere we go, you have to put eyes very closely, you have to monitor people very closely otherwise. We have some dubious characters within the society, so the state is not an exemption…(R-6) |
| 1.6.3 | It’s only this year that we started hearing of probe into the money allocated to HIV/AIDS programme in Nigeria whereby NACA was called by the National Assembly to come and give account but to my surprise, the NACA management admitted that truly truly there was misappropriation because they didn’t have the capacity to do effective supervision of disbursement and monitor projects executed from the…..(R-3) |
| 1.6.4 | If the donors can insist on putting a transparent hopefully an electronic accounting processes in place and government continue to follow that yes the government system should be able to account for these resources (R-12) |
| 1.6.5 | Yes the private sector can play a very good role if [only] they are made to understand very well what is involved and also you know in Nigeria whatever thing you are doing and money is not exchanging hands somebody out there will think that aah after when *you don go chop money na me you go come work [You want me to work for free on something you have been paid for]* so that is where transparency comes in. The government should be able to bring them to round table conference and place all the cards on the table so that everybody will know if they decide to show commitment you show it. If you don’t want to show commitment go, government need to explain all these very well to the private sector and they will give in….(R-2) |
| **1.7** | **Public-Private Partnership** |
| 1.7.1 | Interestingly for Nigeria unlike other countries we have a strong private sector that can be leveraged upon to move supply chain forward…(R-12) |
| 1.7.2 | Like for example, if a program is having a problem with regular report collection. If there is a very powerful, if there is a powerful NGO that can assist in that area at least it will help in some [ways] to reduce reporting issues (R-1) |
| 1.7.3 | …it is not just for partners…we have to look I mean not for donor agencies alone, we have to start looking for individuals who are philanthropists who can provide support you know. (R-8) |
| 1.7.4 | What is the government doing? What happens to all the funds that the government is generating from tax year in year out? (R-3) |
| 1.7.5 | Yes, you know within the country you have, we have some consultancy firms you understand who are into capacity building and in most cases sometimes they get engaged by donors and government alike (R-8) |
| 1.7.6 | Government also needs to move out of the business of trying to run the SC and leverage the private sector competencies (R-10) |
| 1.7.6.1 | finally the private sector in Nigeria is very high so galvanizing the private sector making them more closer to the public health programme without trading off the standards because of course the donors will want to work with the private sector that are more transparent on how they use their finances and also the donors will get value for money it is always number one thing on their agenda (R-12) |
| **1.8** | **Prevention** |
| 1.8.1 | ......you know, we know that the best way of actually treating patients, actually prevention is better, so I think the government should emphasize more on prevention, so that……we don’t have new, new we don’t have proliferation of new patients. So if we can prevent infection of new patients then that that will be better, you know with time…the whole thing will be under control but once we are not making any effort to prevent new infections then automatically we are starting with a failure…..(R-6) |
| **2.0** | **Healthcare Financing** |
| **2.1** | **Domestic Funding** |
| 2.1.1 | …Some level of financial commitment from the government (R-10) |
| 2.1.2.0 | Yes, I think to some extent government made effort especially during the last regime of President Jonathan, ….they tried to create something that is similar to what the United States Presidential Emergency flagged and that…came in the name of Sure P program we have the Sure-P program and the SURE-P Programme was a replica of the United States PEPFAR. It was to provide grants support to institutions that will provide treatment and government pumped a lot of money but whether that is still available today I don’t know…(R-4) |
| 2.1.2.1 | but also government funding also coming up significantly in support of the program (R-12) |
| 2.1.3 | Like the PCRP when it was designed there is this…. public private partnership that we are looking at... Having some structures on ground letting them key into it fully so those were the plans that were on ground but it is not something that government can do alone apart from the donors, private individuals, private firms they need to come in. (R-11) |
| 2.1.4 | Budget line has been approved for the activities of the LMCU and also for the central warehouse and I believe it should be the same thing with the other states too.. (R-8) |
| 2.1.5 | ….Now what is being done now, when the need the entire need of the program is documented and we have commitment from other organizations, we have financial gap that will now require government to tackle. That is one of the ways. Government cannot just say “I have fifty billion take”, No it is going to be wasted….(R-9) |
| 2.1.6 | …Yeah funding is the first one then that one really goes into… in fact that is the root cause of all the other ones because no matter what you design on paper if the funds are not there it will not work, it will not work (R-11) |
| 2.1.7.0 | …..finance is a challenge also em. …but there is a global recession and Nigeria economy presently is in recession so that will be a challenge (R-10) |
| 2.1.7.1 | but the general outcry that resources…you know the decline means we may not be able to set aside enough resources for health as part as a show a commitment... (R-7) |
| 2.1.8 | …You know, that’s why I said that the money is not enough, is not enough because some of the aspects of the programme that were helping to change the course of HIV/AIDS were terminated for instance, there is what we used to call em…..HIV..Voluntary counseling and testing and HCT that is HIV Counselling and Testing, now some years ago in our place these two programs were suspended (R-3) |
| 2.1.9 | No I don’t think so. It will be negligence to the HIV patients in that case. Most of the HIV patients some of them don’t have money… (R-5) |
| 2.1.10 | And then the other part of your question I think…that won’t be an off so that HIV/AIDS [clients] they also pay for their drugs at subsidized rate because…..(R-6) |
| **2.2** | **Bureaucratic Bottlenecks** |
| 2.2.1 | All those things are theoretical ehh it has always been there but pulling out has always been a very difficult thing because most of the approvals are not released (R-2) |
| 2.2.2 | Budget provision is made, gets to the assembly and is slashed down, is appropriated, releases is not on time. Two it is not what is approved that is released…(R-9) |
| **2.3** | **Alternative Funding Sources** |
| 2.3.1 | ….the private sector in the country through things like tax rebates if you put xyz amount of money in public health programme you can get some tax rebates you know that way. You know knocking on the door of the corporate social responsibility of the companies will enable us drive local support drive local finance (R-12) |
| 2.3.2 | … if it is properly managed, mark up from certain percentage of the markup can be set aside for logistics, for commodities movement, for instance in the central medical stores we have a DMA there and we mark up by say 5% for everything for what they buy, out of the 5% a certain percentage could be set aside to support the HIV program as far as commodity management is concerned and we have that in our mind and through peer review we know of a state that is already doing.. the surplus Margin is being used to support the donor funded program (R-7) |
| 2.3.3 | The only thing the government is trying to leverage on is National Health Insurance Scheme (R-9) |
| 2.3.4 | As I said earlier on, challenges of funding, when if there is a advocacy like the one em. Federal Ministry of Health and this iplus consortium did…the other [time] to [X] State. It really affected us, Yes because we were at the house of assembly. We met with the Speaker and the House Committee on Health we met with the Permanent Secretary for Finance, Permanent Secretary for Information, PS Ministry of Health and some relevant Directors and they really promised us. When we were at the defense [it was] something they were already aware so that kind of a thing is needed (R-1) |
| 2.3.5 | I have seen that government government em…need to address even the drug logistics itself because before the emphasis is just buy the drugs for a lot of money invested in procurement but who cares about the logistics, the drug logistic because and those logistics aspect of it can be as expensive as even the procurement….(R-4) |
| 2.3.6 | ….. it will be different now if I say let us go into business if I am also contributing we will have that mutual respect and we will have the say they you see when I tell you this is the direction that we will go that will benefit all of us you will see reasons with me you will want to push back some of your selfish inclinations because you know that I’m also contributing but in a situation where I am not contributing you are the one doing. You will say I don’t care what you want but this is the way I want to go…(R-11) |
| **3.0** | **Health Workforce** |
| **3.1** | **Human Resource for Health, Training and Mentoring, Capabilities and Behavioural Disposition** |
| 3.1.1 | …..[government] made available manpower but we are not using the *oyinbo man* [foreigners] to do all these work in the hither land at least in XX State (R-2) |
| 3.1.2 | Yes in the sense that as far as the support we are receiving from the donor agents or agencies is concerned, they have been able to build the capacity of Nigerians….to take over the ownership (R-7) |
| 3.1.3 | ……pre-service training which the outgone project USAID have started because the curriculum of some university now, this pre-service is already embedded so that people before they graduate, they have understood what they call supply chain. When they get to full practice, it is already part of them…..(R-9) |
| 3.1.4.0 | Some areas at the national level I will say yes but when you go down to the states not yet (R-11) |
| 3.1.4.1 | ……. you know in terms of human resource while they are not in public sector they are in private sector which the government can leverage upon to move the supply chain activities in the country forward. (R-12) |
| 3.1.5 | ……the only thing I can still say is that the government still needs to employ more hands. We have competent pharmacists and other staff. But there is a kind of shortage yeah. Because people have retired, some have even left and the government is still saying they don’t have money. they promised to do something you know this situation [economic recession] will not allow most states to employ…(R-5) |
| 3.1.6.0 | Yes, yes and it is because of the people are not.. this one cannot go in isolation staff attrition. You know when we train somebody today tomorrow he is no more there. Because they are government staff, they can be posted to anywhere (R-1) |
| 3.1.6.1 | The two because we have the Federal Medical Centre in …. here when we trained our doctors here after some….and the remuneration differential they leave the state, For that we don’t complain much, because they still render services within the state, but when they are moving outside the state then it becomes a challenge looking for greener pasture and the only way you can overcome that one is to equate the remuneration (R-7) |
| 3.1.7.0 | But the there is also the area of management, we are happy that we have a lot of organizations that are coming in to help in the areas of management, McKinsey it has come in. Sorry I am mentioning names but I think it is very Mckinsey has come in, ……has come in IBM was here. If you look at all these organizations especially they are tilted towards management either they develop capacity for management but that is because there is a gap in that area because even if you have the, if you take all the money and you procure all the products if there is no proper management, all those things will be wasted. So that it is another challenge (R-4) |
| 3.1.7.1 | Yes. You know there is gap in quantification because we know I know that we don’t have well trained people to do that now it’s not going to be a big problem (R-2) |
| 3.1.8 | …..And em. you know we also have the attitude of our own people where some people get fully committed to their jobs and some are not really very committed and then of course you know we are in a political era so sometimes, some government staff can go wrong, so if they do things, you cannot punish them because someone is knows some governors some ....you understand so these are also some of the challenges (R-8) |
| 3.1.9 | ..Two, on the issue of advocacy, this staff attrition I talked about, we can go and appeal. That we know they are your staff we know they are trained in this area if they want to transfer just fly them. You know, if it is the one in local government A, local government B and is in charge of logistics of HIV/AIDS, if you want to transfer, don’t transfer a new person to that place but rather an already trained one you just swap them.. (R-1) |
| 3.1.10.0 | To overcome the challenges is if government can be made to understand and show interest again in logistics even for the country as a whole by; 1, is to pay the staff, most of them would be able to go all length to provide services from time to time. You know as the----- sometimes the complaints I hear is that, they are not interested or they say they are overwhelmed with their work. So to motivate them one needs to send them for training, they will be motivated to do more you know. Then if we have more hands nobody will tell you he’s overwhelmed again. So the thing is for us at the policy end is to keep presenting it to the government (R-5) |
| 3.1.10.1 | Even those of them who were absorbed, they were not absorbed with the appropriate ….the absorption didn’t take cognizance…..of their qualification and certification. So more like they were forced so to... So in years if you have if you call somebody health assistant and then the peg on his or her career is at Grade level 12 but the person is a graduate who supposed to, who has the right to attain the peak of Grade level 17….(R-3) |
| 3.1.11 | If you are talking about product selection also, the pharmacists are they the best that can fill that position because of their training…(R-3) |
| 3.1.12 | So those and of course the issue of capacity we have said it once is another thing now, more people need to be encouraged to do this work. The greatest problem is that people that [are] working in the HIV program. The greatest challenge is that they work a lot, they work a lot but what do they gain out of it……Certain sectors of the economy because of the strategic importance of what those people are doing they pay them higher…..Because they believe that these people are providing a strategic service so they should extend it to them, drug logistics is a strategic service that demands a lot of documentations, a lot of work because if I am working as a logistician, I should earn more. That is the truth if they do that you will find out that people will develop interest…(R-4) |
| 3.1.13 | Yes for the manpower issue you know there is what we call task shifting. So we have to train the CSS people to become pharmacists. We have to train to the Community Health Extension Workers actually to become Medical Officers so that they will treat patients, those that they can handle they handle them those that they couldn’t you know we have to encourage referrals…..(R-6) |
| 3.1.14 | ……and there is what we call Succession Planning, Mentorship and Succession Planning in place. Like I am in a program now, I work with staff who I draw very close to myself who I give enough opportunities to develop themselves that is in preparation to the time when I will retire from the service but by the time I look back there is no gap….(R-9) |
| 3.1.15 | …..and then em setting clear benchmark for performance and appropriate punishment if those performance targets are not met…(R-12) |
| 3.1.16 | Well 1) I’ll say is government making conscious effort to attract the best brains in the private sector to come into the public sector and giving them the required infrastructure human resource and em.….necessary authority to make the required changes (R-12) |
| 3.1.17 | No you can make it a professional diploma program. You know and then it will be stated that for you to go for the program you must have a degree in so so so and so. In other words, it becomes a professional diploma program or you can even make it a certified professional program…(R-3) |
| **4.0** | **Medical Products, Technologies** |
| **4.1** | **Local Manufacturing** |
| 4.1.1 | so now they wanted the country to own the program. so that the country will now start producing HIV/AIDS commodity and then for opportunistic infections, infection control and so on and so forth (R-6) |
| 4.1.2 | ….. the reason why patient will find it difficult to pay is because the drugs are still being seen as foreign drugs that are imported and therefore their actual cost if I’m permitted to say, it’s a bit artificial. When somebody gives money to somebody to buy the drugs and distribute. So a lot of things may be factored in but if the drugs are available in the market, the informal, just the same way you buy antibiotics… and then there is NHIS cover for antiretroviral drugs ……people…..because you will agree with me the cost of 10 tablets of paracetamol in Nigeria is far far less than the cost in the UK despite the fact that the Nigerian Naira is very low. (R-3) |
| 4.1.2.1 | …..If we have….laudable policies we should be able to, we should be producing these [HIV] commodities in-country……there are instances we run out of the commodities but if they are locally produced we will be able to have these commodities. These are some of the commitments (R-7) |
| **4.2** | **Unification and Integration** |
| 4.2.1 | HIV in particular, now as am talking to you before we used to have commodities coming from different directions going to different directions you know…that was what happened in the country so there was a drive by the program from the federal ministry of health, you know to unify, of course with the support of partners as well to unify the supply chain so irrespective of where the commodities are coming from and irrespective of where they are meant for, they come through the same channel, they come through you understand that is why they called it unification (R-8) |
| 4.2.2 | …that is why it is thought [to be] cost effective to have all the supply chain integrated so that the five disease programs are currently being integrated, one HIV program, the ATM, A-AIDS T-Tuberculosis, M- Malaria, Reproductive health and Vaccines for immunizations (R-9) |
| 4.2.3 | Yes so that’s why we are hoping that eem with the NSCIP put in place, we have a common tool for all the programs....to help reduce the burden of [on] [healthcare] worker[s] (R-2) |
| 4.2.4 | …. So integrate HIV/AIDS care with the normal conventional health care programe because the more you create a special clinic for HIV/AIDS care, the worst the stigmatization. So government needs to work on that (R-2) |
| **5.0** | **Information and research** |
| **5.1** | **Data availability, Data Quality and Data for Action** |
| 5.1.1 | No as a department, we didn’t know where the commodities [for other program areas] were kept. Because of the integration and establishment of the LMCU we now know………We cost we cost these commodities that are taken to the facilities and are used so we know their value which makes us.....that’s what… these are tools that are used to remind my superiors, my principals, why…we need to know and we need to plan for sustainability because we now know the cost of these commodities…….We also conduct QSSR and QSSR enable us to know where commodities are, the value of these commodities and expiry dates of these commodities in the facilities which allows for inter-facility re-distribution and minimizing loses that can arise from expiry. (R-7) |
| 5.1.2 | Well I think yes I must give credit to SCMS [for data visibility] and I think that is what they were doing. They were building; they were working towards a system that allows visibility…..(R-4) |
| 5.1.2.1 | We hope that the next budget, by the time we submit a lot of quarterly stock status reports, commissioner will be able to see what we are doing. (R-5) |
| 5.1.3 | Evidence of some level of plan, evidence of some level of consciousness on the part of the government to now ask for a plan and em. efforts to align plans with available funding no matter how small the fund is these are measures of progress that we when you go to the ministry now the ministry request have you seen the report of the national quantification to initiate a procurement even if they are not going to procure all that is required that’s a step in the right direction.. (R-12) |
| 5.1.4 | we are having this integrated program now so that the LMCU [is] in place then we would be able to overcome the problem of proper data because if you don’t have accurate data, reliable data it means that all you are doing is rubbish so we have a lot of challenges but I believe that we are going to overcome these challenges…we are trying to harmonize and harness the people involved (R-2) |
| 5.1.5 | Does Nigerian government know the number of people being admitted for malaria in a month? Does Nigeria government know the number of people being treated for onchocerciasis? Does Nigeria government know how many people are reporting malaria infections while on HIV treatment? We don’t. Do we have the data on our monthly consumption of antimalarial drugs….(R-3) |
| 5.1.6 | Upgrade the data capturing system this idea of em…of em….getting data from paper work is completely outdated, it’s outdated completely, that is the first thing so that you will have what we call reliable data, reliable data if you saying 3.1 it is 3.1…(R-3) |
| **6.0** | **Service Delivery** |
| **6.1** | **Infrastructure** |
| 6.1.1 | ….for the entire country now, we are moving towards public and private partnership which is being done in other parts of the world, so that it is now a joint responsibility, government has a role to play, private sector have a role to play, so that collaboration is expected to bring a very fruitful and good impact on the supply chain. Now we have warehouse in a box with M.O.U signed with the government to ensure PPP. So those are kind of broad sustainability plan which is kick start, infact it is kick starting now from warehouse in a box in Abuja here….(R-9) |
| 6.1.2 | ……government staff that are working within that warehouse of course they will enjoy the support of capacity building and a lot of other things from partners, so we have such similar storage structures in each geo political zone and because of that the Federal Medical Store has been decongested instead of having commodities coming in from there when they come directly from the port, instead been kept at Federal Medical Stores in Lagos if they are meant for Northwest....[they] go to Northwest, Northeast will go to Northeast like that….(R-8) |
| 6.1.2.1 | …Then another thing is the clinic [warehouse] - in- the box. The clinic [warehouse] in the box, is em…came through USAID, USAID has really helped…It is the biggest structure so far that I have seen in Nigeria. I was there recently and I saw what they have done warehouse yes sorry warehouse in a box and we have one in Abuja and we have another one in Lagos. (R-4) |
| 6.1.3 | Of course some infrastructures they gave us some….em….computers they gave us some chairs and tables though we still need more actually.. (R-1) |
| 6.1.4 | …It’s just to provide like vehicles that will take them. Like in …[X State], if you move from… [XXXX] to ….[YYYYY] it’s a nightmare the roads are not good and all that. So if government can make available some cars like the Hilux® vans and all that….(R-2) |
| **6.2** | **Access** |
| 6.2.1 | ……and then of recent we were at, we went round to see how we can ...err…activate some new sites and so we went for site assessment. For PMTCT and then HTC sites ….everything was on the bill of the state government so I think the country is having some impact (R-6) |
| 6.2.1.1 | I can say there is still so much left to be desired especially in the area of commodity procurement and also ensuring universal coverage, because there is still left to…be desired in terms of scale up of these services because in some of the states the facilities providing the services are not enough compared to the population….(R-8) |
| 6.2.2 | …..though HIV has actually made a lot of progress in terms of people accepting treatment (R-8) |
| 6.2.3 | ….some private hospitals that are also being used for the work of em.. HIV…..em…we have a lot of private hospitals around (R-2) |
| 6.2.4 | …Recently there was this push that government says test and treat and then that means the government want to put on treatment about 3.1 million people compared to less than 1.5 million that we currently have. From the budget we know that the government does not have that plan….(R-3) |
