# Supplementary figures and images for: Country ownership and sustainability of Nigeria’s HIV/AIDS Supply Chain System: qualitative perceptions of progress, challenges and prospects
Source: J Pharm Policy Pract. 2018 Sep 10;11:21. doi: 10.1186/s40545-018-0148-8 (PMC6130083; doi:10.1186/s40545-018-0148-8)

**Ethics Approval**


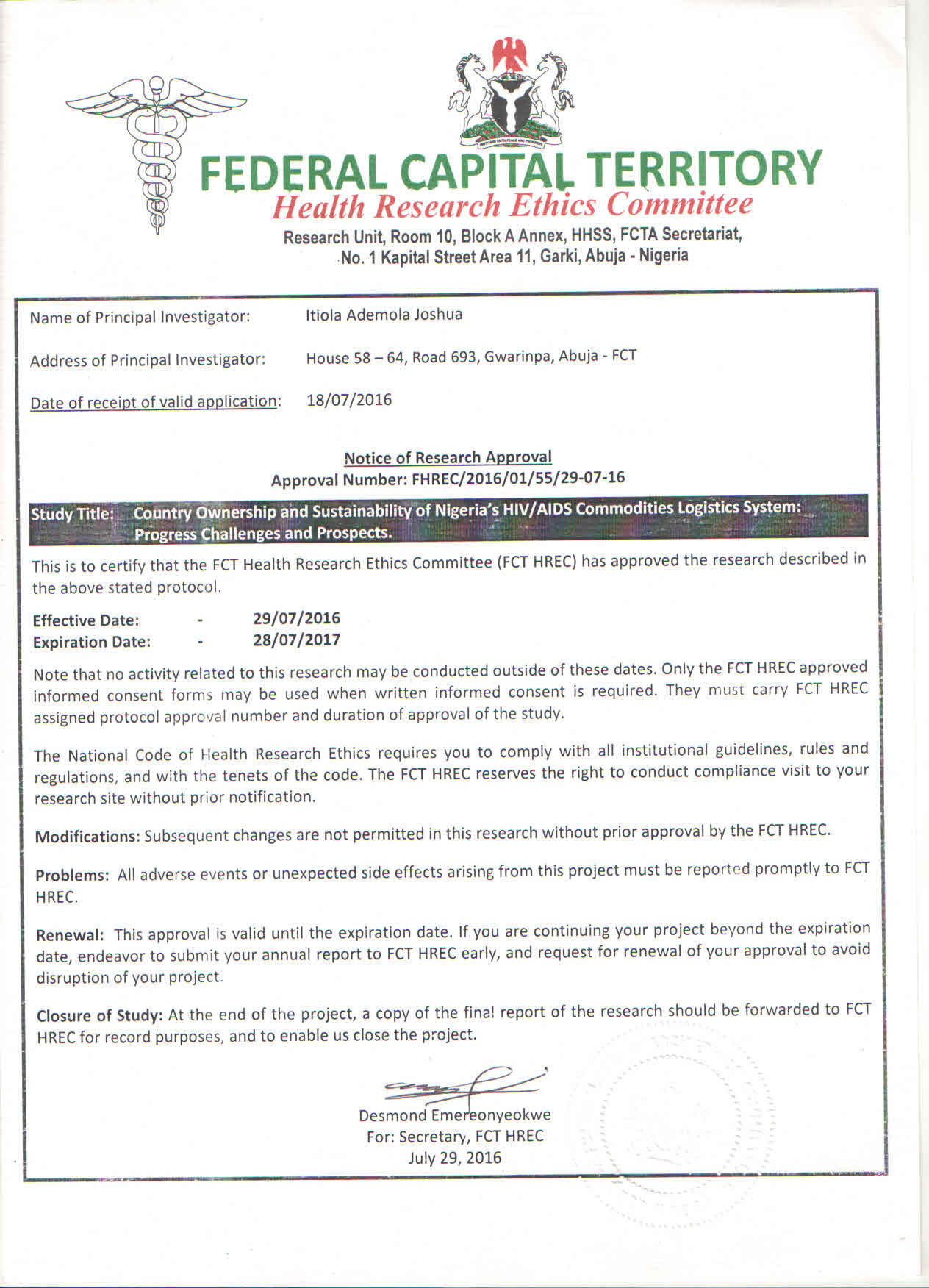

Supplement: Supplementary file 5 — Ethics Approval. (DOCX 223 kb) [file 40545_2018_148_MOESM5_ESM.docx]
